# A Theoretical Study on Static Gas Pressure Measurement via Circular Non-Touch Mode Capacitive Pressure Sensor

**DOI:** 10.3390/s24165314

**Published:** 2024-08-16

**Authors:** Ji Wu, Xiao-Ting He, Jun-Yi Sun

**Affiliations:** 1 School of Civil Engineering, Chongqing University, Chongqing 400045, China; 202116131099t@cqu.edu.cn (J.W.); hexiaoting@cqu.edu.cn (X.-T.H.); 2 Key Laboratory of New Technology for Construction of Cities in Mountain Area (Chongqing University), Ministry of Education, Chongqing 400045, China

**Keywords:** capacitive pressure sensor, static gas pressure, large deflection, analytical solution, numerical calibration

## Abstract

A circular non-touch mode capacitive pressure sensor can operate in both transverse and normal uniform loading modes, but the elastic behavior of its movable electrode plate is different under the two different loading modes, making its input–output analytical relationships between pressure and capacitance different. This suggests that when such a sensor operates, respectively, in transverse and normal uniform loading modes, the theory of its numerical design and calibration is different, in other words, the theory for the transverse uniform loading mode (available in the literature) cannot be used as the theory for the normal uniform loading mode (not yet available in the literature). In this paper, a circular non-touch mode capacitive pressure sensor operating in normal uniform loading mode is considered. The elastic behavior of the movable electrode plate of the sensor under normal uniform loading is analytically solved with the improved governing equations, and the improved analytical solution obtained can be used to mathematically describe the movable electrode plate with larger elastic deflections, in comparison with the existing two analytical solutions in the literature. This provides a larger technical space for developing the circular non-touch mode capacitive pressure sensors used for measuring the static gas pressure (belonging to normal uniform loading).

## 1. Introduction

A capacitive pressure sensor uses a variable capacitor to detect pressure and convert the detected pressure into capacitance as an electrical signal output [1,2,3,4,5]. The variable capacitor consists of a pair of fixed and movable electrode plates, where the fixed electrode plate is a rigid conductive plate coated by a thin layer of insulator, the movable electrode plate is usually a flexible conductive membrane that is initially flat and peripherally fixed, and the medium filled in the gap between the insulator layer and the movable electrode plate is generally air [6,7,8,9,10]. When pressure is applied onto the surface of the initially flat conductive membrane, the membrane will deflect elastically toward the fixed electrode plate and produce a non-parallel movement due to its fixed outer edge. Such a non-parallel movement makes the variable capacitor change from an initial parallel plate capacitor to a non-parallel plate capacitor, and the capacitance of the variable capacitor increases due to the decrease in the gap between the two electrode plates. Therefore, when the variable capacitor is at rest, the value of the applied pressure corresponds one-to-one to the value of the capacitance, and after this one-to-one correspondence (i.e., the analytical relationship between the input pressure and the output capacitance) is known, then the applied pressure can be determined by measuring the capacitance. This is how a capacitive pressure sensor works. The capacitive pressure sensor is usually called a touch mode capacitive pressure sensor when its movable electrode plate (the conductive membrane) is in contact with the insulator layer [11,12,13,14,15] and is called a non-touch mode capacitive pressure sensor before its movable electrode plate (the conductive membrane) is in contact with the insulator layer [16,17,18,19,20].

Obviously, essential to the numerical design and calibration of a measurement system using such capacitive pressure sensors is the ability to obtain the pressure–capacitance analytical relationship of the variable capacitor used. The capacitance value of the non-parallel plate variable capacitor at rest depends on the non-parallel distance between the fixed and movable electrode plates, more specifically, on the spatial geometry of the deflected movable electrode plate (conductive membrane) under the applied pressure, which is determined by the elastic deflections at the points on the deflected conductive membrane. Therefore, the elastic behavior of the initially flat conductive membrane under the applied pressure has to be exactly solved analytically, so as to use the obtained analytical solution of deflection to exactly derive the capacitance expression of the non-parallel plate capacitor [21,22,23,24,25]. However, since membranes are prone to large elastic deflections under pressure, the mathematical equations governing their elastic behaviors often show strong geometric nonlinearity [26,27,28,29,30]. Therefore, analytical solutions for membrane problems are available in few cases, because these somewhat intractable nonlinear equations usually present serious analytical difficulties [31,32,33,34,35]. This has also been a key scientific and technical problem to be solved in the research and development of such capacitive pressure sensors.

Relatively speaking, the analytical solutions of the large deflection elastic behavior of the rectangular membranes are often much more difficult to be obtained, while that of the circular membranes are relatively easy to be obtained, due to the fact that the large deflection deformations of circular membrane structures or structural components are always axisymmetric, reducing the level of difficulty of analytically solving these large deflection deformations [36,37,38,39,40]. Naturally, a circular capacitive pressure sensor uses a circular conductive membrane as the movable electrode plate of its variable capacitor and can operate in either touch mode or non-touch mode. Not only that, but also the circular movable electrode plate (circular conductive membrane) can be loaded in different ways; for example, it can elastically behave under either uniformly distributed transverse loads or uniformly distributed normal loads. Obviously, the two different loading modes will result in two different elastic behaviors of the circular movable electrode plate (circular conductive membrane); that is, when the uniformly distributed transverse loads has the same intensity as the uniformly distributed normal loads, the spatial geometries of the circular movable electrode plate (circular conductive membrane) are different under the two loading modes, thus making the non-parallel plate variable capacitor between the fixed and movable electrode plates have two different capacitance values under the two loading modes. In other words, for the same circular capacitive pressure sensor, the pressure–capacitance analytical relationship that the sensor operates in the transverse uniform loading mode is different from the one that the sensor operates in the normal uniform loading mode. Therefore, for the same circular capacitive pressure sensor, if the capacitance value measured when the sensor operates in transverse uniform loading mode is equal to that measured when the sensor operates in normal uniform loading mode, then the load intensity that the sensor operates with in the transverse uniform loading mode is not equal to the load intensity that the sensor operates with in the normal uniform loading mode. This implies that for the same circular capacitive pressure sensor, the theory of numerical design and calibration that is used to measure uniformly distributed transverse loads is different from the one that is used to measure uniformly distributed normal loads; that is, the two different theories cannot be used interchangeably. However, in the existing literature, it is difficult to find reports on the measurement of uniformly distributed normal loads via such circular capacitive pressure sensors.

In this paper, a theoretical study on the measurement of the static gas pressure (belonging to uniformly distributed normal loads) through a circular non-touch mode capacitive pressure sensor is presented for the first time. Obviously, the pressure exerted on the circular movable electrode plate (the circular conductive membrane) of the sensor due to a high-speed moving gas or liquid should belong to uniformly distributed transverse loads, while the pressure exerted on the membrane via a static gas or liquid belongs to uniformly distributed normal loads. Therefore, all the theories of using circular non-touch mode capacitive pressure sensors for the pressure measurement, which are presented by regarding the circular movable electrode plate under the pressure as an initially flat, peripherally fixed circular membrane under the action of uniformly distributed transverse loads (as reported in [41,42]), can only be used to measure the pressure exerted on the circular movable electrode plate due to the high-speed moving gas or liquid, and cannot be used to measure the pressure exerted on the circular movable electrode plate via the static gas or liquid. However, the theory of using a circular non-touch mode capacitive pressure sensor for the static gas pressure measurement has yet to be established.

## 2. Materials and Methods

A measurement system for static gas pressure via a circular non-touch mode capacitive pressure sensor involves the treatment of two key scientific and technical issues: one is the derivation of the capacitance of the sensor under the static gas pressure (the derivation of the analytical relationship of input pressure and output capacitance of the sensor), and the other is the analytical solution to the elastic behavior of the circular movable electrode plate of the sensor under the static gas pressure (which is necessary for the calculation of the capacitance of the sensor).

### 2.1. The Derivation of the Input–Output Analytical Relationship of the Sensor

**Figure 1:**
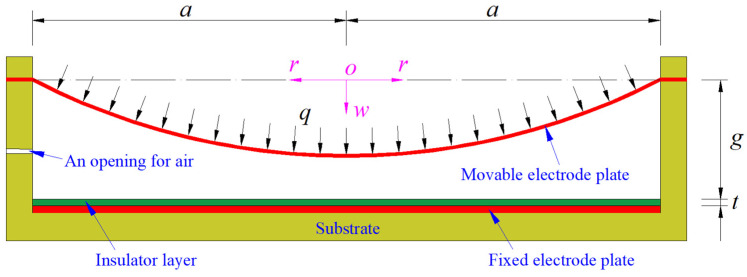
A circular non-touch mode capacitive pressure sensor under the static gas pressure *q*.

A circular capacitive pressure sensor is assumed to operate in non-touch mode after it is subjected to a static gas pressure *q*, as shown in Figure 1, where the dash-dotted line denotes the geometric middle plane of the initially flat movable electrode plate that has not yet been subjected to the action of the static gas pressure *q*, *a* denotes the radius of the initially flat movable electrode plate, *t* denotes the thickness of the insulator layer coating on the fixed electrode plate, *g* denotes the initial parallel gap between the initially flat movable electrode plate and the insulator layer, and the polar plane (*r*, *φ*) of the introduced cylindrical coordinate system (*r*, *φ*, *w*) is located in the plane in which the geometric middle plane is located, coordinate origin *o* is coincident with the centroid of the geometric middle plane, and the radial, circumferential, and transverse coordinates are *r*, *φ*, and *w*, respectively; also, *w* denotes the deflection (transverse displacement) of the movable electrode plate under the static gas pressure *q*. The movable electrode plate is generally made of a conductive membrane and the fixed electrode plate is generally made of a flat rigid conductive thin plate fixed to the substrate.

When the static gas pressure *q* is applied, the initially flat movable electrode plate will axisymmetrically deflect towards the fixed electrode plate. This makes the variable capacitor between the movable and fixed electrode plates change from a parallel plate capacitor to a non-parallel plate capacitor. The spatial geometry of the deflected movable electrode plate under the static gas pressure *q* can be expressed as *w*(*r*) in the cylindrical coordinate system (*r*, *φ*, *w*); that is, it is only related to the radial coordinate *r* and not to the angle coordinate *φ*, due to the axisymmetric deflection of the movable electrode plate. The variable capacitor between the two electrode plates can be regarded as consisting of two capacitors in series: one is the non-parallel plate capacitor between the insulator layer and the movable electrode plate, and the other is the parallel plate capacitor between the fixed electrode plate and the insulator layer. Therefore, the total capacitance *C* of the variable capacitor between the two electrode plates, i.e., the capacitance *C* of the sensor, may be written as

(1) $$\frac{1}{C}=\frac{1}{C_{1}}+\frac{1}{C_{2}},$$

where *C*_1_ refers to the capacitance of the parallel plate capacitor between the fixed electrode plate and the insulator layer, and *C*_2_ refers to the capacitance of the non-parallel plate capacitor between the insulator layer and the movable electrode plate. If the vacuum permittivity is denoted by *ε*_0_, the relative permittivity of the insulator layer is denoted by *ε_r_*_1_, and the relative permittivity of the air is denoted by *ε_r_*_2_, then the capacitance *C*_1_ may be written as

(2) $$C_{1}=\frac{\varepsilon_{0}\varepsilon_{r1}\pi a^{2}}{t},$$

and the capacitance *C*_2_ may be written as [41]

(3) $$C_{2}=\int_{0}^{a}\int_{0}^{2\pi }\varepsilon_{0}\varepsilon_{r2}\frac{r}{g-w(r)}d\varphi dr=2\pi \varepsilon_{0}\varepsilon_{r2}\int_{0}^{a}\frac{r}{g-w(r)}dr.$$

From Equations (1)–(3), the total capacitance *C* of the variable capacitor between the movable and fixed electrode plates may finally be written as

(4) $$C=\frac{C_{1}C_{2}}{C_{1}+C_{2}}=\frac{2\pi \varepsilon_{0}\varepsilon_{r1}\varepsilon_{r2}a^{2}\int_{0}^{a}\frac{r}{g-w(r)}dr}{\varepsilon_{r1}a^{2}+2\varepsilon_{r2}t\int_{0}^{a}\frac{r}{g-w(r)}dr}.$$

Therefore, the capacitance *C* of the circular non-touch mode capacitive pressure sensor under the static gas pressure *q* can be determined with Equation (4), where the deflection function *w*(*r*) depends on the magnitude of the applied static gas pressure *q*. Since the capacitance *C* of the circular non-touch mode capacitive pressure sensor corresponds one-to-one to the applied static gas pressure *q*, Equation (4) is actually the analytical relationship of the input pressure as an independent variable and the output capacitance as a dependent variable (the pressure–capacitance (*p*–*C*) analytical relationship) of the sensor. Therefore, due to the one-to-one *p*–*C* analytical relationship, the applied static gas pressure *q* can be determined by measuring the capacitance *C* of the sensor as long as the deflection function *w*(*r*) under the static gas pressure *q* can be obtained. This is the idea behind the development of this circular non-touch mode capacitive pressure sensor.

However, the deflection function *w*(*r*) can only be obtained by analytically solving the elastic behavior of the circular movable electrode plate under the static gas pressure *q*. In addition, the hardware design of this circular non-touch mode capacitive pressure sensor also requires the stress solution *σ*(*r*) to check the material strength of the movable electrode plate being used. So, the analytical solution to the elastic behavior of the circular movable electrode plate under the static gas pressure *q* is necessary for the design of this circular non-touch mode capacitive pressure sensor used for measuring the load intensity of the applied static gas pressure *q*. 

### 2.2. The Analytical Solution to the Elastic Behavior of the Movable Electrode Plate of the Sensor

**Figure 2:**
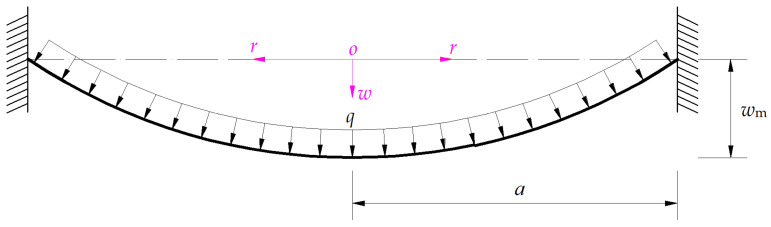
A profile along the diameter of the deflected circular membrane under the uniformly distributed normal load *q*.

The movable electrode plate of a circular non-touch mode capacitive pressure sensor is assumed to use a circular conductive membrane. Therefore, the elastic behavior of the circular movable electrode plate under the static gas pressure *q* may be regarded mechanically as a large deflection problem of an initially flat, peripherally fixed circular membrane with radius *a*, thickness *h*, Poisson’s ratio *ν*, and Young’s modulus of elasticity *E* under the action of uniformly distributed normal load *q*, as shown in Figure 2, where *w*_m_ denotes the maximum elastic deflection of the axisymmetrically deformed circular membrane under the uniformly distributed normal load *q*. Obviously, if the maximum pressure to be measured via the sensor is denoted as *q*_m_, then the maximum deflection *w*_m_ of the circular membrane under *q*_m_ determines the initial parallel gap *g* in Figure 1 and its maximum stress *σ*_m_ determines the material strength when the movable electrode plate (conductive membrane) is selected.

**Figure 3:**
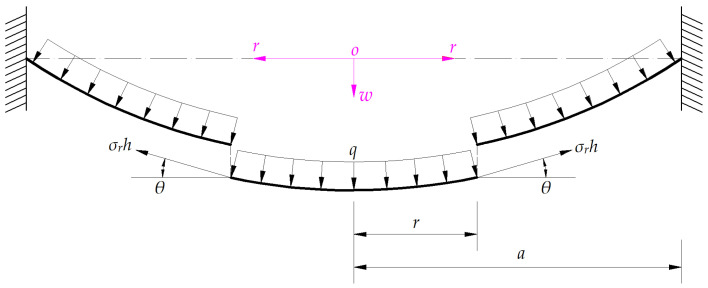
A free body of radius *r* (0 ≤ *r* ≤ *a*) under the joint action of the uniformly distributed normal load *q* and the membrane force *σ*_r_*h*.

A free body of radius *r* (0 ≤ *r* ≤ *a*) is cut from the central portion of the deflected circular membrane in Figure 2, to study its static problem of equilibrium, as shown in Figure 3, where *σ_r_* is the radial stress at *r*, *σ_r_h* is the membrane force acting on the boundary *r*, and *θ* denotes the slope angle at *r*. Therefore, in the vertical direction perpendicular to the polar plane (*r*, *φ*), there is a downward force *πr*^2^*q* and an upward force 2*πrσ_r_h*sin*θ*. Then, from the condition of the resultant force in the vertical direction being equal to zero, the out-of-plane equilibrium equation can be written as

(5) $$\pi r^{2}q-2\pi r\sigma_{r}h\sin\theta =0,$$

where

(6) $$\sin\theta =1/\sqrt{1+1/\tan^{2}\theta }=1/\sqrt{1+1/{\left(-dw/dr\right)}^{2}}.$$

Substituting Equation (6) into Equation (5) yields

(7) $$rq\sqrt{1+1/{\left(-dw/dr\right)}^{2}}-2\sigma_{r}h=0.$$

Fichter [43] presented an in-plane equilibrium equation, which is suitable for the large deflection problem of the initially flat, peripherally fixed circular membrane under uniformly distributed normal loads and is expressed as

(8) $$\frac{d}{dr}(r\sigma_{r}h)-\sigma_{t}h-qr\frac{dw}{dr}=0,$$

where *σ_t_* denotes the circumferential stress. Equation (8) is the first, and also the only, in-plane equilibrium equation that takes into account the action of the normal load *q* on the circular membrane in the horizontal direction parallel to the polar plane (*r*, *φ*) and is still used as the in-plane equilibrium equation here.

The classical radial geometric equation has been improved by Lian et al. [34], since it is only applicable to the case where the deflection of the membrane is not too large (generally, a deflection greater than 1/5 of the membrane thickness is called a large deflection). The improved radial geometric equation can be used for membrane problems that allow the membrane deflection to be larger than the membrane thickness, or even several times larger, and is expressed as [34]

(9) $$e_{r}=\sqrt{{\left(1+\frac{du}{dr}\right)}^{2}+{\left(-\frac{dw}{dr}\right)}^{2}}-1,$$

where *u* and *e_r_* denote the radial displacement and radial strain, respectively. The circumferential geometric equation is expressed as [34]

(10) $$e_{t}=\frac{u}{r},$$

where *e_t_* denotes the circumferential strain.

The two-dimensional stress–strain relationships follow the generalized Hooke’s law and are expressed as

(11) $$\sigma_{r}=\frac{E}{1-\nu^{2}}(e_{r}+\nu e_{t})$$

and

(12) $$\sigma_{t}=\frac{E}{1-\nu^{2}}(e_{t}+\nu e_{r}).$$

Equations (7)–(12) are six equations governing the elastic behavior of the circular membrane in Figure 2, which are described by stresses *σ_r_* and *σ_t_*, strains *e_r_* and *e_t_*, and displacements *u* and *w*. The boundary conditions for solving these six governing equations are

(13) $$\frac{dw}{dr}=0\;\mathrm{at}\;r=0,$$

(14) $$e_{t}=0\;\mathrm{at}\;r=a$$

and

(15) w=0 at r=a.

Let us eliminate strains *e_r_* and *e_t_* from Equations (9)–(12) first. Substituting Equations (9) and (10) into Equations (11) and (12) yields

(16) $$\sigma_{r}=\frac{E}{1-\nu^{2}}\{{\left[{\left(1+\frac{du}{dr}\right)}^{2}+{\left(\frac{dw}{dr}\right)}^{2}\right]}^{1/2}-1+\nu \frac{u}{r}\}$$

and

(17) $$\sigma_{t}=\frac{E}{1-\nu^{2}}\{\frac{u}{r}+\nu {\left[{\left(1+\frac{du}{dr}\right)}^{2}+{\left(\frac{dw}{dr}\right)}^{2}\right]}^{1/2}-\nu \}.$$

Equation (16) multiplied by *v* minus Equation (17) yields

(18) $$\frac{u}{r}=\frac{1}{E}(\sigma_{t}-\nu \sigma_{r}).$$

Therefore, from Equations (10) and (18), the boundary condition Equation (14) can be expressed in terms of stress conditions

(19) $$\sigma_{t}-\nu \sigma_{r}=0\;\mathrm{at}\;r=a.$$

Further, substituting the *u* in Equation (18) into Equation (16) yields

(20) $${\left(\frac{1}{E}\sigma_{r}+1-\frac{\nu }{E}\sigma_{t}\right)}^{2}-{\left\{1+\frac{d}{dr}[\frac{1}{E}r(\sigma_{t}-\nu \sigma_{r})]\right\}}^{2}-{\left(-\frac{dw}{dr}\right)}^{2}=0.$$

Equations (7), (8) and (20) are three equations for the solution of *σ_r_*, *σ_t_*, and *w*.

Let us introduce the following dimensionless variables

(21) $$Q=\frac{qa}{Eh},\;W=\frac{w}{a},\;S_{r}=\frac{\sigma_{r}}{E},\;S_{t}=\frac{\sigma_{t}}{E},\;x=\frac{r}{a}$$

and transform Equations (7), (8), (20), (13), (15) and (19) into

(22) $$(4{S_{r}}^{2}-x^{2}Q^{2}){\left(-\frac{dW}{dx}\right)}^{2}-x^{2}Q^{2}=0,$$

(23) $$\frac{d}{dx}(xS_{r})-S_{t}-Qx\frac{dW}{dx}=0,$$

(24) $${\left(1+S_{r}-\nu S_{t}\right)}^{2}-{\left(1+S_{t}+x\frac{dS_{t}}{dx}-\nu S_{r}-\nu x\frac{dS_{r}}{dx}\right)}^{2}-{\left(-\frac{dW}{dx}\right)}^{2}=0,$$

(25) $$\frac{dW}{dx}=0\;\mathrm{at}\;x=0,$$

(26) W=0 at x=1

and

(27) $$S_{t}-\nu S_{r}=0\;\mathrm{at}\;x=1.$$

In order to use the power series method to solve Equations (22)–(24), *S_r_*(*x*), *S_t_*(*x*), and *W*(*x*) have to be expanded into the power series of the *x*, i.e.,

(28) $$S_{r}(x)=\sum_{i=0}^{\infty }b_{i}x^{i},$$

(29) $$S_{t}(x)=\sum_{i=0}^{\infty }c_{i}x^{i}$$

and

(30) $$W(x)=\sum_{i=0}^{\infty }d_{i}x^{i}.$$

The recursion formulas for the power series coefficients *b_i_*, *c_i_*, and *d_i_* can be obtained by substituting Equations (28)–(30) into Equations (22)–(24) and letting all the coefficient sums of the *x* to the same power be equal simultaneously to zero, as shown in Appendix A, where, when *i* = 1, 3, 5,…, the coefficients *b_i_*, *c_i_*, and *d_i_* are constantly equal to zero, and when *i* = 2, 4, 6,…, they can be expressed into the polynomials with regard to the coefficient *b*_0_, and *c*_0_ ≡ *b*_0_. Therefore, there are only two undetermined constants, that is, the coefficients *b*_0_ and *d*_0_, which can be determined by using the boundary conditions Equations (27) and (26).

From Equations (28) and (29), the boundary condition Equation (27) yields

(31) $$\sum_{i=0}^{\infty }c_{i}-\nu \sum_{i=0}^{\infty }b_{i}=0.$$

Since the coefficients *b_i_* and *c_i_* can be expressed into the polynomials with regard to the coefficient *b*_0_ and *c*_0_ ≡ *b*_0_, Equation (31) contains only one variable *b*_0_. Therefore, the undetermined constant *b*_0_ can be determined by Equation (31). Further, from Equation (30), the boundary condition Equation (26) yields

(32) $$d_{0}=-\sum_{i=1}^{\infty }d_{i}.$$

Since the coefficients *d_i_* (*i* = 1, 3, 5, …) are constantly equal to zero and the coefficients *d_i_* (*i* = 2, 4, 6, …) can be determined with the known *b*_0_, the undetermined constant *d*_0_ can be determined by using Equation (32).

Therefore, for a concrete problem in which the parameter values of *a*, *h*, *E*, *ν*, and *q* are known beforehand, all the coefficients *b_i_*, *c_i_*, and *d_i_* (*i* = 0, 1, 2, 3, …) can be determined, so all the expressions of the radial stress *σ_r_*, circumferential stress *σ_t_*, and transverse displacement (deflection) *w* can be determined. From Equations (21) and (28)–(30), the dimensional forms of *σ_r_*, *σ_t_*, and *w* can be written as

(33) $$\sigma_{r}(r)=E\sum_{i=0}^{\infty }b_{i}{\left(\frac{r}{a}\right)}^{i},$$

(34) $$\sigma_{t}(r)=E\sum_{i=0}^{\infty }c_{i}{\left(\frac{r}{a}\right)}^{i}$$

and

(35) $$w(r)=a\sum_{i=0}^{\infty }d_{i}{\left(\frac{r}{a}\right)}^{i}.$$

As for the expressions of the radial displacement *u*, radial strain *e_r_*, and circumferential strain *e_t_*, with the known expressions of *σ_r_*, *σ_t_*, and *w*, they can be easily derived from Equations (18), (10) and (9). It is not necessary to derive them here, as the design of a circular non-touch mode capacitive pressure sensor requires only the analytical expressions of the deflection *w* and stresses *σ_r_* and *σ_t_* (used for determining the maximum stress *σ*_m_ to check the strength of the membrane material used).

## 3. Results and Discussion

### 3.1. Difference between Elastic Behaviors of Transversely and Normally Loaded Membrane

The movable electrode plate of a circular non-touch mode capacitive pressure sensor is assumed to use a circular conductive membrane with Poisson’s ratio *v* = 0.45, Young’s modulus of elasticity *E* = 3.05 MPa, radius *a* = 70 mm, and thickness *h* = 0.3 mm.

**Figure 4:**
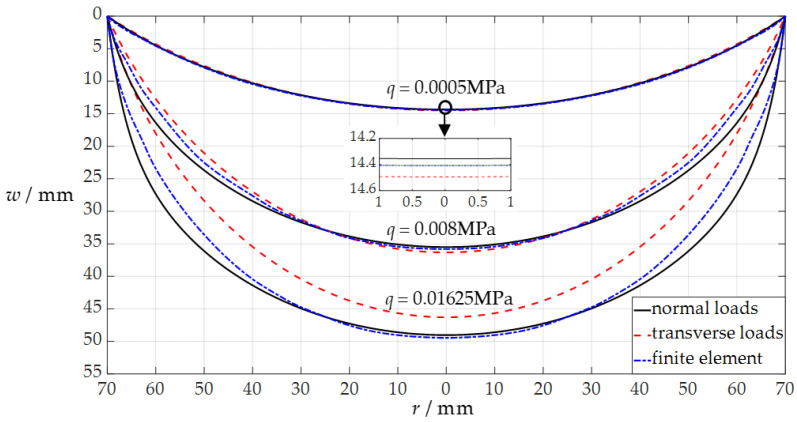
The variation of *w* with *r* when *q* takes the values 0.0005 MPa, 0.008 MPa, and 0.01625 MPa.

Suppose that the uniformly distributed normal loads that are applied to the circular membrane are *q* = 0.0005 MPa, *q* = 0.008 MPa, and *q* = 0.01625 MPa, respectively, then it can be obtained from Equation (21) that *Q* = 0.038251 for *q* = 0.0005 MPa, *Q* = 0.612022 for *q* = 0.008 MPa, and *Q* = 1.243169 for *q* = 0.01625 MPa. Therefore, the undetermined constants *b*_0_ and *d*_0_ can be determined by using Equations (31) and (32) and the recursion formulas for the power series coefficients *b_i_*, *c_i_*, and *d_i_* in Appendix A, which are *b*_0_ = 0.056631 and *d*_0_ = 0.205102 for *q* = 0.0005 MPa, *b*_0_ = 0.540343 and *d*_0_ = 0.507193 for *q* = 0.008 MPa, and *b*_0_ = 1.025862 and *d*_0_ = 0.700458 for *q* = 0.01625 MPa, respectively. Finally, from Equation (35), the deflection curves *w*(*r*) of the circular conductive membrane (the movable electrode plate) under *q* = 0.0005 MPa, *q* = 0.008 MPa, and *q* = 0.01625 MPa can be obtained, as shown by the three solid lines in Figure 4. The three dashed lines in Figure 4 represent the cases where the same circular conductive membrane is subjected to the uniformly distributed transverse loads of *q* = 0.0005 MPa, *q* = 0.008 MPa, and *q* = 0.01625 MPa, respectively, which are obtained by using the analytical solution presented in [34], where *b*_0_ = 0.053005 and *d*_0_ = 0.207083 for *q* = 0.0005 MPa, *b*_0_ = 0.400731 and *d*_0_ = 0.518867 for *q* = 0.008 MPa, and *b*_0_ = 0.710715 and *d*_0_ = 0.661283 for *q* = 0.01625 MPa. The three dash–dotted lines in Figure 4 represent the results of the finite element simulation (using the four-node quadrilateral membrane elements (M3D4R) in ABAQUS (2024) software) when the same circular conductive membrane is subjected to the static gas pressures of *q* = 0.0005 MPa, *q* = 0.008 MPa, and *q* = 0.01625 MPa, respectively.

The difference between the analytical solutions presented in [34] and in Section 2.2 in this paper lies only in the different in-plane equilibrium equations adopted by each, while the other governing equations (the out-of-plane equilibrium equations, geometric equations, and physical equations) are completely the same. Comparing the in-plane equilibrium equation used in Section 2.2 in this paper (Equation (8) in this paper) with that used in [34] (Equation (4) in [34]), it is easy to find that there is an extra term *qr*d*w*/d*r* in the in-plane equilibrium equation adopted in this paper. This additional term *qr*d*w*/d*r* takes into account the in-plane component of the uniformly distributed normal loads acting on the deflected membrane. Therefore, the difference between the solid and dotted lines in Figure 4 increases as the load *q* increases, showing that the two analytical solutions for the uniformly distributed normal and transverse loads agree quite closely for lightly loaded membranes and diverge slowly as the load intensifies. This implies that the analytical solution of an initially flat, peripherally fixed circular membrane under uniformly distributed transverse loads is not suitable for describing the elastic behavior of an initially flat, peripherally fixed circular membrane under uniformly distributed normal loads; that is, the analytical solution of transverse uniform loading is not suitable for the numerical design and calibration of a circular non-touch mode capacitive pressure sensor used for static gas pressure measurement. In the following section, this issue will be discussed further from the point of view of the analytical relationship of the input pressure as an independent variable and the output capacitance as a dependent variable (the pressure–capacitance (*p*–*C*) analytical relationship) of the sensor.

### 3.2. Difference between the p–C Relationships of the Sensor under Normal and Transverse Loads 

**Figure 5:**
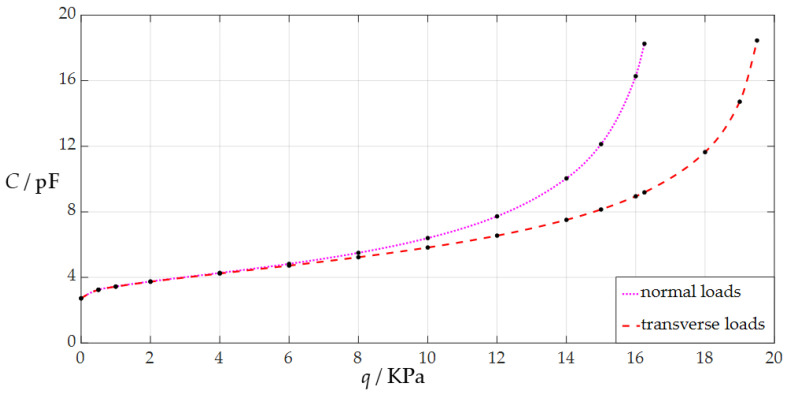
The variation in the output capacitance *C* with the input pressure *q* for the sensor with *a* = 70 mm, *h* = 0.3 mm, *t* = 0.1 mm, *E* = 3.05 MPa, *ν* = 0.45, and *g* = 50 mm, where the dotted line represents the pressure–capacitance (*p*–*C*) analytical relationship when the sensor is used for measuring normal loads and the dashed line represents the *p*–*C* analytical relationship when the sensor is used for measuring transverse loads.

The circular conductive membrane, which is used as the movable electrode plate of a circular non-touch mode capacitive pressure sensor in Section 3.1, is still used here, but the uniformly distributed normal load *q* will gradually increase from 0.0005 MPa. When the sensor is subjected to the uniformly distributed normal load *q*, the numerical calculation results of the undetermined constants *b*_0_ and *d*_0_, maximum membrane deflection *w*_m_, and capacitance *C* are listed in Table A1, where the maximum membrane deflection is expressed as *w*_m_ = *ad*_0_ (see Equation (35)), the capacitance *C* is determined via Equation (4), the initial parallel gap between the initially flat movable electrode plate and the insulator layer is *g* = 50 mm, the thickness of the insulator layer is *t* = 0.1 mm, the vacuum permittivity is *ε*_0_ = 8.854 × 10^−3^ pF/mm, the relative permittivity of the insulator layer is *ε_r_*_1_ = 2.5 (polystyrene), and the air relative permittivity is *ε_r_*_2_ = 1.00053. When the sensor is subjected to the uniformly distributed transverse load *q*, all the numerical calculation results are listed in Table A2, which are calculated by using the analytical solution presented in [34]. Figure 5 is the analytical relationship of the input pressure as an independent variable and the output capacitance as a dependent variable (the pressure–capacitance (*p*–*C*) analytical relationship) drawn with the numerical values of the pressure *q* and capacitance *C* in Table A1 and Table A2, where the dotted line represents the *p*–*C* analytical relationship when the sensor is used for measuring normal loads and the dashed line represents the *p*–*C* analytical relationship when the sensor is used for measuring transverse loads.

The difference between the pressure–capacitance (*p*–*C*) analytical relationships of the normally and transversely loaded sensor with *a* = 70 mm, *h* = 0.3 mm, *t* = 0.1 mm, *E* = 3.05 MPa, *ν* = 0.45, and *g* = 50 mm, i.e., the difference between the dotted and dashed lines in Figure 5, reconfirms the conclusion drawn in Section 3.1: the analytical solution of transverse loading is not suitable for the numerical design and calibration of a circular non-touch mode capacitive pressure sensor used for static gas pressure measurement.

### 3.3. The Beneficial Effect of the Improved Analytical Solution in Section 2.2

So far, in the existing literature there are only two analytical solutions for the large deflection problem of a peripherally fixed and initially flat circular membrane subjected to uniformly distributed normal loads, and they are the analytical solutions provided by Fichter [43] and He et al. [44], respectively. Fichter [43] originally dealt with this large deflection problem and provided an analytical solution of the problem, which is the first analytical solution of the large deflection problem of circular membranes under normal loading. Based on the analytical solution provided by Fichter [43], He et al. improved the governing equation describing the out-of-plane equilibrium of the deformed membrane (i.e., the so-called out-of-plane equilibrium equation), and provided an improved analytical solution for the large deflection problem [44]. In this paper, based on the improved analytical solution provided by He et al. [44], we further improve the governing equation describing the relationship between the radial displacement and radial strain (i.e., the so-called radial geometric equation), and provide a newer analytical solution for the large deflection problem.

**Figure 6:**
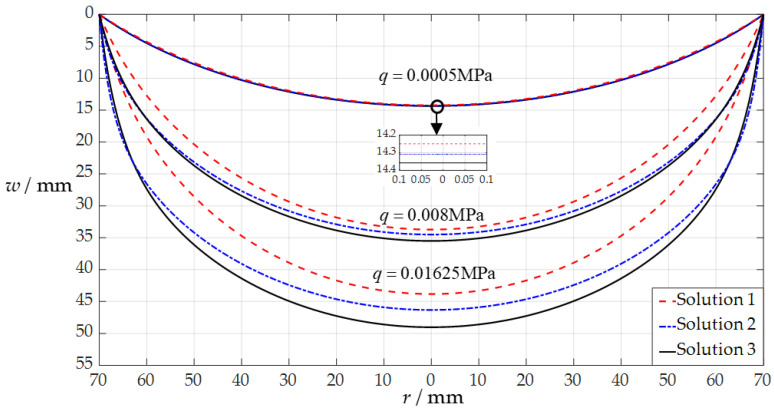
The variation of *w* with *r* when *q* takes the values 0.0005 MPa, 0.008 MPa, and 0.01625 MPa, where “Solution 1” refers to the result calculated using the analytical solution presented in [43], “Solution 2” refers to the result calculated using the analytical solution presented in [44], and “Solution 3” refers to the result calculated using the analytical solution presented in Section 2.2 in this paper.

The difference between the out-of-plane equilibrium equations before and after the improvement is reflected in the difference between Equation (2) in [43] and Equation (3) in [44] (which is identical to Equation (7) in this paper), while the difference between the radial geometric equations before and after the improvement is reflected in the difference between Equation (6) in [43] (or Equation (5) in [44]) and Equation (9) in this paper. Essentially, these differences are the results of giving up the assumption that the rotation angle of the membrane is very small. Therefore, these analytical solutions obtained with the assumption of a small rotation angle of the membrane are not suitable for applications with relatively large deflections, as shown in Figure 6, where “Solution 1” refers to the calculation result via the analytical solution presented in [43], “Solution 2” refers to the calculation result via the analytical solution in [44], and “Solution 3” refers to the calculation result via the analytical solution in this paper.

It can be seen from Figure 6 that the three analytical solutions agree quite closely for lightly loaded membranes but diverge slowly as the uniformly distributed normal load *q* intensifies, showing that the improvements made have achieved the expected positive effect.

### 3.4. An Example Illustration of Numerical Design and Calibration

In this section, an example is provided to illustrate how to use the analytical solution obtained in Section 2.1 to numerically design and calibrate a circular non-touch mode capacitive pressure sensor used for measuring static gas pressure. The sensor is assumed to be used for measuring the static gas pressure of *q* = 0~0.016 MPa, and its design starts with a preliminary designation of design parameters (materials and dimensions). The relevant design parameters that need to be preliminarily specified include the radius *a* of the sensor, the relative permittivity *ε_r_*_1_ and thickness *t* of the insulation layer, and the Poisson’s ratio *v* and Young’s modulus of elasticity *E* of the circular conductive membrane (the movable electrode plate). The thickness *h* of the circular conductive membrane and the initial parallel gap *g* between the initially flat movable electrode plate and the insulator layer are quantities that need to be determined according to the maximum static gas pressure *q* = 0.016 MPa and the yield strength *σ*_y_ of the preliminarily specified conductive membrane.

The radius *a* of the sensor is usually preliminarily specified according to the size of the space in which the sensor is placed, and is assumed to be specified as *a* = 70 mm. 

The insulator layer of the sensor is assumed to be polyethylene. Its relative permittivity is *ε_r_*_1_ = 2.5 and its thickness is assumed to be specified as *t* = 0.1 mm.

The movable electrode plate of this sensor is assumed to use a circular conductive membrane with radius *a* = 70 mm, Poisson’s ratio *v* = 0.45, and Young’s modulus of elasticity *E* = 3.05 MPa. 

In addition, the air relative permittivity is *ε_r_*_2_ = 1.00053, and the vacuum permittivity is *ε*_0_ = 8.854 × 10^−3^ pF/mm. Suppose that the yield strength of the preliminarily specified conductive membrane is about *σ*_y_ = 4.5 MPa.

The thickness *h* of the preliminarily specified conductive membrane can be determined as follows. First, let *h* takes an arbitrary value (suppose *h* = 0.3 mm). Then, with *q* = 0.016 MPa, *a* = 70 mm, *h* = 0.3 mm, *v* = 0.45, and *E* = 3.05 MPa, the undetermined constants *b*_0_ and *d*_0_ can be calculated using Equations (21), (31) and (32) and the recursion formulas for the power series coefficients *b_i_*, *c_i_*, and *d_i_* in Appendix A (the calculated results are *b*_0_ = 1.011422 and *d*_0_ = 0.694655). Further, with the known *b*_0_ and *d*_0_, the radial stress *σ_r_* and circumferential stress *σ_t_* can be calculated using Equations (33) and (34), and the maximum membrane stress *σ*_m_ takes the largest of the maximum value of the radial stress *σ_r_* and the maximum value of the circumferential stress *σ_t_* (the calculated result is that the maximum value of the radial stress *σ_r_* is the largest so *σ*_m_ = 3.085 MPa). If the maximum stress *σ*_m_ in this calculation is much less than 0.7*σ*_y_, then *h* = 0.3 mm does not meet the material strength requirements, and a slightly reduced value of *h* is needed to recalculate the maximum stress *σ*_m_ until the material strength requirement is met. If the maximum stress *σ*_m_ in this calculation is much greater than 0.7*σ*_y_, then *h* = 0.3 mm also does not meet the material strength requirements, and a slightly increased value of *h* is needed to recalculate the maximum stress *σ*_m_ until the material strength requirement is met. If the maximum stress *σ*_m_ in this calculation is close to 0.7*σ*_y_, then the arbitrary assumption of *h* = 0.3 mm meets the material strength requirement (since *σ*_m_ = 3.085 MPa, *σ*_y_ = 4.5 MPa, and *σ*_m_ ≤ 0.7*σ*_y_; therefore, *h* = 0.3 mm can meet the material strength requirement).

After the membrane thickness *h* is determined to be *h* = 0.3 mm, the initial parallel gap *g* between the initially flat movable electrode plate and the insulator layer can be determined as follows. First, the maximum deflection *w*_m_ of the circular membrane with *a* = 70 mm, *h* = 0.3 mm, *v* = 0.45, and *E* = 3.05 MPa under the action of the uniformly distributed normal load *q* = 0.016 MPa can be calculated using Equation (35), and the calculated result is *w*_m_ = *ad*_0_ = 70 × 0.694655 = 48.62585 (mm). Therefore, the initial parallel gap *g* may be roughly taken as *g* = 50 mm temporarily; that is, the initial parallel gap *g* should be greater than and close to the maximum deflection *w*_m_ = 48.62585 mm. Finally, the maximum stress *σ*_m_ when the maximum deflection *w*_m_ reaches *w*_m_ = 50 mm needs to be further calculated; the calculated result is that when the maximum deflection *w*_m_ reaches *w*_m_ = 50 mm, the uniformly distributed normal load *q* is about *q* = 0.016839 MPa, the corresponding undetermined constants are *b*_0_ = 1.059829 and *d*_0_ = 0.714170, and the maximum value of the radial stress *σ_r_* is maximum and is about 3.232 MPa, so *σ*_m_ = 3.232 MPa < 0.8*σ*_y_. Therefore, the initial parallel gap *g* = 50 mm is appropriate.

After the above numerical design of the sensor hardware is completed, the numerical calibration of the sensor can be achieved as follows. Since the mechanism of using a circular non-touch mode capacitive pressure sensor to measure the static gas pressure *q* (as shown in Figure 1) is to detect the static gas pressure *q* by measuring the capacitance *C* of the sensor to which the static gas pressure *q* is applied, then such a static gas pressure measurement system requires an analytical relationship of the input capacitance as an independent variable and the output pressure as a dependent variable (the capacitance–pressure (*C*–*p*) analytical relationship), rather than the analytical relationship of the input pressure as an independent variable and the output capacitance as a dependent variable (the pressure–capacitance (*p*–*C*) analytical relationship) shown in Equation (4).

**Figure 7:**
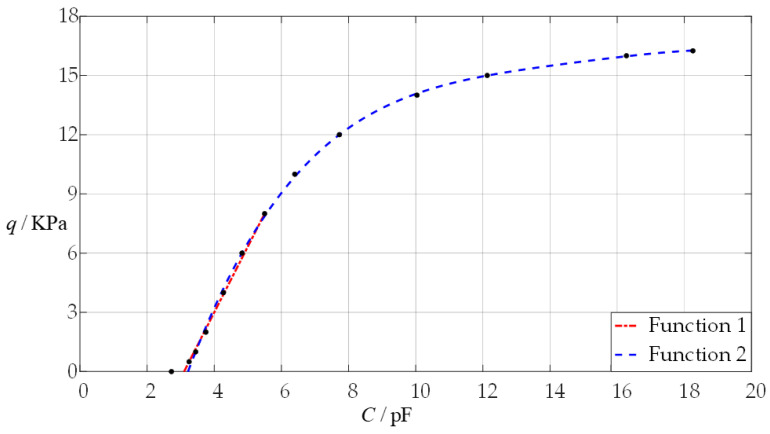
The capacitance–pressure (*C*–*p*) relationship of the sensor with *a* = 70 mm, *h* = 0.3 mm, *t* = 0.1 mm, *E* = 3.05 MPa, *ν* = 0.45, *g* = 50 mm, and its least-squares data fitting, where “Function 1” is fitted by a straight line, and “Function 2” is fitted by a quartic curve, which are listed in Table 1.

Obviously, due to the complexity of Equation (4) (see the recursion formulas for the power series coefficients *b_i_*, *c_i_*, and *d_i_* in Appendix A), the required analytical relationship of the input capacitance as an independent variable and the output pressure as a dependent variable (the capacitance–pressure (*C*–*p*) analytical relationship) cannot be derived directly from Equation (4). It can only be obtained through least-squares data fitting based on a large number of numerical calculation values for the capacitance *C*. To this end, the pressure *q* has to start from a smaller value to calculate the numerical values of the undetermined constants *b*_0_ and *d*_0_, maximum deflection *w*_m_, maximum stress *σ*_m_, and capacitance *C*, as shown in Table A3. Based on the numerical calculation values of the pressure *q* and capacitance *C* in Table A3, the required *C–p* analytical relationship is shown in Figure 7 and is fitted with a straight line and a curve via the least-square method as shown in Table 1.

The linear capacitance–pressure (*C*–*q*) analytical relationship of “Function 1” in Figure 7 and Table 1 has a simple analytical expression and is therefore suitable for analog techniques, while the nonlinear capacitance–pressure (*C*–*q*) analytical relationship of “Function 2” in Figure 7 and Table 1 has a complex analytical expression and is therefore only suitable for digital techniques. However, the variation ranges of the pressure *q* and capacitance *C* in the *C*–*q* analytical relationships often need to be adjusted, including the ratio of pressure *q* to capacitance *C*, to meet the use requirements and technical needs. This adjustment can be achieved by changing some or just one of the design parameters, such as the radius *a*, thickness *h*, Young’s modulus of elasticity *E*, and Poisson’s ratio *v* of the circular membrane, etc. In this case, it is necessary to know the effect of changing design parameters on the *C*–*q* analytical relationships, which is addressed in the following section.

### 3.5. Influence of Changing Design Parameters on the C–q Analytical Relationships

In this section, we will analyze the influence of changing some design parameters of a circular non-touch mode capacitive pressure sensor used for measuring static gas pressure on the capacitance–pressure (*C*–*q*) analytical relationship of the sensor, such as the radius *a*, thickness *h*, Young’s modulus of elasticity *E*, and Poisson’s ratio *v* of the circular membrane, the thickness *t* of the insulator layer, and the initial parallel gap *g* between the initially flat movable electrode plate and the insulator layer.

#### 3.5.1. Influence of Changing Membrane Thickness *h* on *C–q* Relationships

The design parameters in this section are as follows. The Poisson’s ratio still takes *v* = 0.45, the Young’s modulus of elasticity still takes *E* = 3.05 MPa, the radius of the circular membrane still takes *a* = 70 mm, the thickness of the insulator layer still takes *t* = 0.1 mm, and the initially parallel gap still takes the value *g* = 50 mm, while the thickness *h* of the circular membrane takes 0.3 mm, 0.6 mm, and 0.9 mm, respectively. In addition, the vacuum permittivity is *ε*_0_ = 8.854 × 10^−3^ pF/mm, the air relative permittivity is *ε_r_*_2_ = 1.00053, and the insulator layer still uses polystyrene, and its relative permittivity is *ε_r_*_1_ = 2.5. 

**Figure 8:**
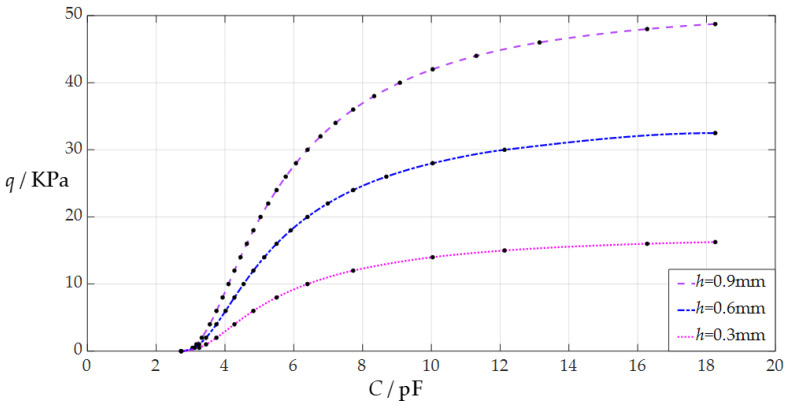
The effect of changing the membrane thickness *h* on the *C*–*q* relationships when *a* = 70 mm, *g* = 50 mm, *t* = 0.1 mm, *ν* = 0.45, and *h* takes the values 0.3 mm, 0.6 mm, and 0.9 mm, respectively.

When the uniformly distributed normal load *q* takes different values, the numerical calculation results of the undetermined constants *b*_0_ and *d*_0_, maximum membrane deflection *w*_m_, maximum membrane stress *σ*_m_, and capacitance *C* are listed in Table A3 for *h* = 0.3 mm, in Table A4 for *h* = 0.6 mm, and in Table A5 for *h* = 0.9 mm. Figure 8 shows the effect of changing the membrane thickness *h* on the *C*–*q* analytical relationships. As can be seen from Figure 8, the increase in the membrane thickness *h* does not change the range of the input capacitance *C* but can increase the range of the output pressure *q*. The degree of influence when increasing the membrane thickness *h* on the range increase for the output pressure *q* can be defined as a percentage of the two relative increments of the output pressure *q* and membrane thickness *h*. The relative increment of the membrane thickness *h* is equal to (0.9 mm − 0.3 mm)/0.3 mm = 200%, and from Table A3 and Table A5, the relative increment of the maximum output pressure *q* is equal to (48.75 KPa − 16.25 KPa)/16.25 KPa = 200%; so, the degree of influence when increasing the membrane thickness *h* on the range increase for the output pressure *q* is 200%/200% = 100%; that is, a 1% increase in the membrane thickness *h* can increase the maximum output pressure *q* by 1%.

#### 3.5.2. Influence of Changing Young’s Modulus of Elasticity *E* on *C*–*p* Relationships

The design parameters in this section are *a* = 70 mm, *h* = 0.3 mm, *v* = 0.45, *t* = 0.1 mm, *g* = 50 mm, *ε*_0_ = 8.854 × 10^−3^ pF/mm, *ε_r_*_2_ = 1.00053, *ε_r_*_1_ = 2.5, and the Young’s modulus of elasticity *E* takes the values 3.05 MPa, 5 MPa and 8 MPa, respectively.

**Figure 9:**
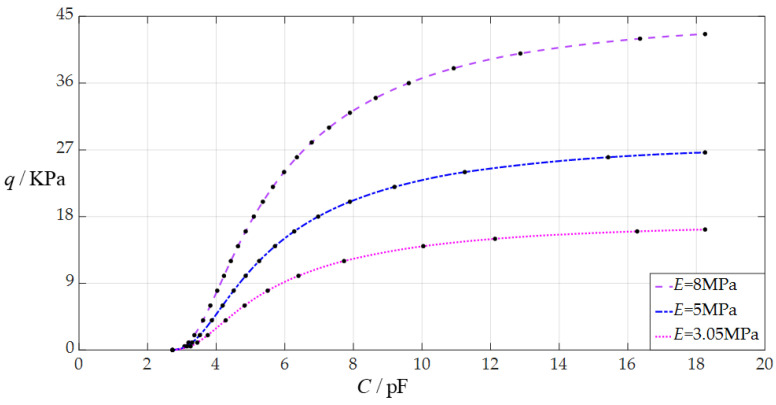
The effect of changing the Young’s modulus of elasticity *E* on the *C*–*q* relationships when *a* = 70 mm, *g* = 50 mm, *h* = 0.3 mm, *t* = 0.1 mm, *ν* = 0.45, and *E* takes the values 3.05 MPa, 5 MPa, and 8 MPa, respectively.

When the uniformly distributed normal load *q* takes different values, the numerical calculation results of the undetermined constants *b*_0_ and *d*_0_, maximum membrane deflection *w*_m_, maximum membrane stress *σ*_m_, and capacitance *C* are listed in Table A3 for *E* = 3.05 MPa, in Table A6 for *E* = 5 MPa, and in Table A7 for *E* = 8 MPa. Figure 9 shows the effect of changing Young’s modulus of elasticity *E* on the *C*–*q* analytical relationships. As can be seen from Figure 9, the increase in Young’s modulus of elasticity *E* does not change the range of the input capacitance *C* but can increase the range of the output pressure *q*. The degree of influence when increasing Young’s modulus of elasticity *E* on the range increase for the output pressure *q* can be defined as a percentage of the two relative increments of the output pressure *q* and Young’s modulus of elasticity *E*. The relative increment of Young’s modulus of elasticity *E* is equal to (8 MPa − 3.05 MPa)/3.05 MPa = 162.30%, and from Table A3 and Table A7, the relative increment of the maximum output pressure *q* is equal to (42.623 KPa − 16.25 KPa)/16.25 KPa = 162.30%; so, the degree of influence when increasing the Young’s modulus of elasticity *E* on the range increase for the output pressure *q* is 162.30%/162.30% = 100%; that is, a 1% increase in the Young’s modulus of elasticity *E* can increase the maximum output pressure *q* by 1%.

#### 3.5.3. Influence of Changing Poisson’s Ratio *v* on *C*–*q* Relationships

The design parameters in this section are *a* = 70 mm, *h* = 0.3 mm, *E* = 3.05 MPa, *t* = 0.1 mm, *g* = 50 mm, *ε*_0_ = 8.854 × 10^−3^ pF/mm, *ε_r_*_2_ = 1.00053, *ε_r_*_1_ = 2.5, and the Poisson’s ratio *v* takes the values 0.45, 0.3, and 0.15, respectively.

**Figure 10:**
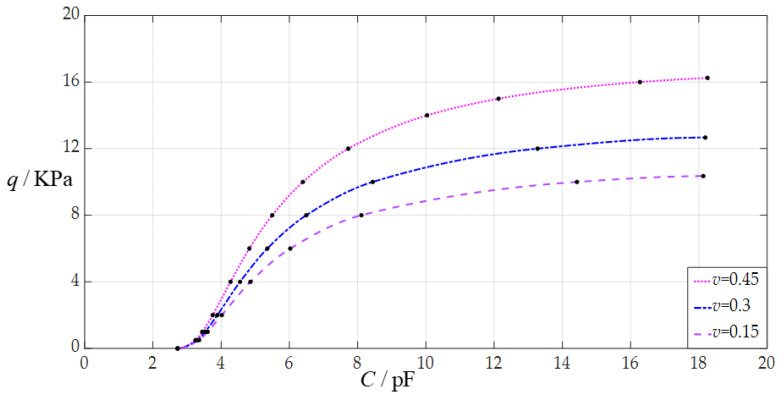
The effect of changing the Poisson’s ratio *v* on the *C*–*q* relationships when *a* = 70 mm, *g* = 50 mm, *h* = 0.3 mm, *t* = 0.1 mm, *E* = 3.05 MPa, and *v* takes 0.45, 0.3 and 0.15, respectively.

When the uniformly distributed normal load *q* takes different values, the numerical calculation results of the undetermined constants *b*_0_ and *d*_0_, maximum membrane deflection *w*_m_, maximum membrane stress *σ*_m_, and capacitance *C* are listed in Table A3 for *v* = 0.45, in Table A8 for *v* = 0.3, and in Table A9 for *v* = 0.15. Figure 10 shows the effect of changing Poisson’s ratio *v* on the *C*–*q* analytical relationships. As can be seen from Figure 10, the increase in the Poisson’s ratio *v* does not change the range of the input capacitance *C* but can increase the range of the output pressure *q*. The degree of influence when increasing the Poisson’s ratio *v* on the range increase for the output pressure *q* can be defined as a percentage of the two relative increments of the output pressure *q* and Poisson’s ratio *v*. The relative increment of the Poisson’s ratio *v* is equal to (0.45 − 0.15)/0.15 = 200%, and from Table A3 and Table A9, the relative increment of the maximum output pressure *q* is equal to (16.25 KPa − 10.353 KPa)/10.353 KPa = 56.96%; so, the degree of influence when increasing the Poisson’s ratio *v* on the range increase for the output pressure *q* is 56.96%/200% = 28.48%; that is, a 1% increase in the Poisson’s ratio *v* can increase the maximum output pressure *q* by 0.2848%.

#### 3.5.4. Influence of Changing Insulator Layer Thickness *t* on *C*–*q* Relationships

The design parameters in this section are *a* = 70 mm, *h* = 0.3 mm, *E* = 3.05 MPa, *v* = 0.45, *g* = 50 mm, *ε*_0_ = 8.854 × 10^−3^ pF/mm, *ε_r_*_2_ = 1.00053, *ε_r_*_1_ = 2.5, and the insulator layer thickness *t* takes the values 0.1 mm, 5 mm, and 10 mm, respectively.

**Figure 11:**
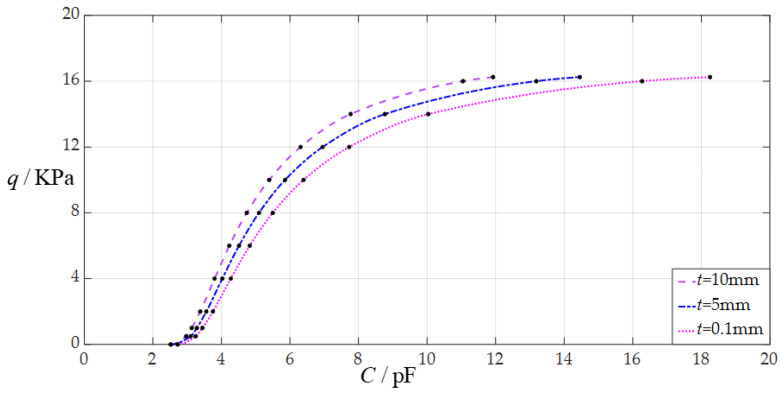
The effect of changing the insulator layer thickness *t* on the *C*–*q* relationships when *a* = 70 mm, *g* = 50 mm, *h* = 0.3 mm, *E* = 3.05 MPa, *ν* = 0.45, and *t* takes the values 0.1 mm, 5 mm, and 10 mm, respectively.

When the uniformly distributed normal load *q* takes different values, the numerical calculation results of the undetermined constants *b*_0_ and *d*_0_, maximum membrane deflection *w*_m_, maximum membrane stress *σ*_m_, and capacitance *C* are listed in Table A3 for *t* = 0.1 mm, in Table A10 for *t* = 5 mm, and in Table A11 for *t* = 10 mm. Figure 11 shows the effect of changing the insulator layer thickness *t* on the *C*–*q* analytical relationships. As can be seen from Figure 11, the decrease in the insulator layer thickness *t* does not change the range of the output pressure *q* but can increase the range of the input capacitance *C*. The degree of influence when decreasing the insulator layer thickness *t* on the range increase for the input capacitance *C* can be defined as a percentage of the two relative increments of the input capacitance *C* and insulator layer thickness *t*. The relative decrement of the insulator layer thickness *t* is equal to (0.1 mm − 10 mm)/10 mm = −99%, and from Table A3 and Table A11, the relative increment of the maximum input capacitance *C* is equal to (18.254 pF − 11.926 pF)/1.926 pF = 53.06%; so, the degree of influence when decreasing the insulator layer thickness *t* on the range increase for the input capacitance *C* is 53.06%/99% = 53.60%; that is, a 1% decrease in the insulator layer thickness *t* can increase the maximum input capacitance *C* by 0.536%.

#### 3.5.5. Influence of Changing Circular Membrane Radius *a* on *C*–*q* Relationships

The design parameters in this section are *h* = 0.3 mm, *E* = 3.05 MPa, *v* = 0.45, *t* = 0.1 mm, *g* = 50 mm, *ε*_0_ = 8.854 × 10^−3^ pF/mm, *ε_r_*_2_ = 1.00053, *ε_r_*_1_ = 2.5, and the radius *a* of the circular membrane takes the values 70 mm, 80 mm, and 90 mm, respectively.

**Figure 12:**
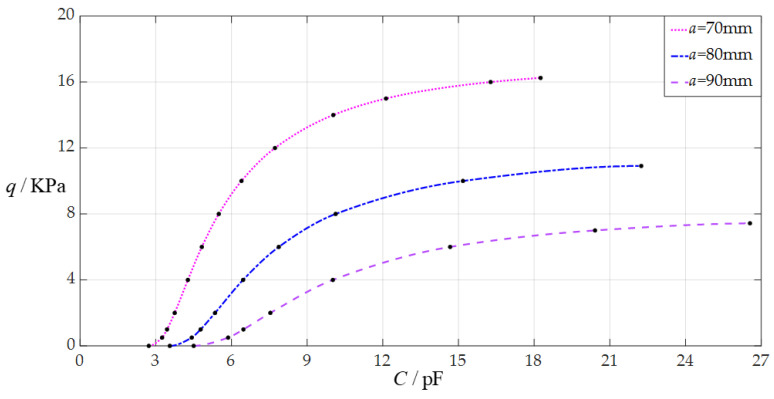
The effect of changing the circular membrane radius *a* on *C*–*q* relationships when *g* = 50 mm, *h* = 0.3 mm, *t* = 0.1 mm, *E* = 3.05 MPa, *ν* = 0.45, and *a* takes the values 70 mm, 80 mm, and 90 mm, respectively.

When the uniformly distributed normal load *q* takes different values, the numerical calculation results of the undetermined constants *b*_0_ and *d*_0_, maximum membrane deflection *w*_m_, maximum membrane stress *σ*_m_, and capacitance *C* are listed in Table A3 for *a* = 70 mm, in Table A12 for *a* = 80 mm, and in Table A13 for *a* = 90 mm. Figure 12 shows the effect of changing the radius *a* of the circular membrane on the *C*–*q* analytical relationships. As can be seen from Figure 12, the increase in the radius *a* of the circular membrane can decrease the range of the output pressure *q* and increase the range of the input capacitance *C*. The degree of influence when increasing the radius *a* of the circular membrane on the range decrease for the output pressure *q* can be defined as a percentage of the two relative increments of the output pressure *q* and radius *a* of the circular membrane. The degree of influence when increasing the radius *a* of the circular membrane on the range increase for the input capacitance *C* can be defined as a percentage of the two relative increments of the input capacitance *C* and radius *a* of the circular membrane. The relative increment of the radius *a* of the circular membrane is equal to (90 mm − 70 mm)/70 mm = 28.57%, and from Table A3 and Table A13, the relative decrement of the maximum output pressure *q* is equal to (7.435 KPa − 16.25 KPa)/16.25 KPa = −54.25%, and the relative increment of the maximum input capacitance *C* is equal to (26.553 pF − 18.254 pF)/18.254 pF = 45.46%; so, the degree of influence when increasing the radius *a* of the circular membrane on the range decrease for the output pressure *q* is 54.25%/28.57% = 189.88%, and the degree of influence when increasing the radius *a* of the circular membrane on the range increase for the input capacitance *C* is 45.46%/28.57% = 159.12%; that is, a 1% increase in the radius *a* of the circular membrane can decrease the maximum output pressure *q* by 1.8988% and can increase the maximum input capacitance *C* by 1.5912%.

#### 3.5.6. Influence of Changing Initially Parallel Gap *g* on *C–q* Relationships

The design parameters in this section are *a* = 70 mm, *h* = 0.3 mm, *E* = 3.05 MPa, *v* = 0.45, *t* = 0.1 mm, *ε*_0_ = 8.854 × 10^−3^ pF/mm, *ε_r_*_2_ = 1.00053, *ε*_r1_ = 2.5, and the initially parallel gap *g* takes the values 50 mm, 55 mm, and 60 mm, respectively.

**Figure 13:**
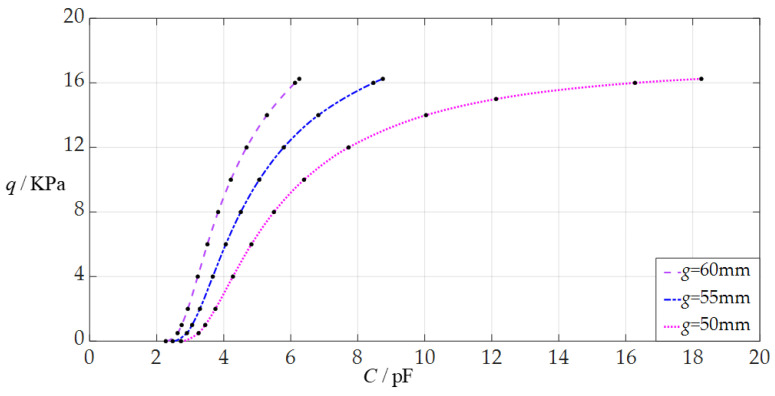
The effect of changing the initially parallel gap *g* on *C*–*q* relationships when *a* = 70 mm, *h* = 0.3 mm, *t* = 0.1 mm, *E* = 3.05 MPa, *ν* = 0.45, and *g* takes the values 50 mm, 55 mm, and 60 mm, respectively.

When the uniformly distributed normal load *q* takes different values, the numerical calculation results of the undetermined constants *b*_0_ and *d*_0_, maximum membrane deflection *w*_m_, maximum membrane stress *σ*_m_, and capacitance *C* are listed in Table A3 for *g* = 50 mm, in Table A14 for *g* = 55 mm, and in Table A15 for *g* = 60 mm. Figure 13 shows the effect of changing the initial parallel gap *g* on the *C*–*q* analytical relationships. As can be seen from Figure 13, the decrease in the initially parallel gap *g* does not change the range of the output pressure *q* but can increase the range of the input capacitance *C*. The degree of influence when decreasing the initially parallel gap *g* on the range increase for the input capacitance *C* can be defined as a percentage of the two relative increments of the input capacitance *C* and initially parallel gap *g*. The relative decrement of the initially parallel gap *g* is equal to (50 mm – 60 mm)/60 mm = −16.67%, and from Table A3 and Table A15, the relative increment of the maximum input capacitance *C* is equal to (18.254 pF − 6.255 pF)/6.255 pF = 191.83%; so, the degree of influence when decreasing the initially parallel gap *g* on the range increase for the input capacitance *C* is 191.83%/16.67% = 1150.75%; that is, a 1% decrease in the initially parallel gap *g* can increase the maximum input capacitance *C* by 11.5075%.

## 4. Concluding Remarks

In this paper, a theoretical study on the measurement of static gas pressure via a circular non-touch mode capacitive pressure sensor is presented for the first time. From this study, the following conclusions can be drawn.

The circular non-touch mode capacitive pressure sensor can be used for measuring both uniformly distributed transverse and uniformly distributed normal loads. However, the input–output analytical relationship between pressure and capacitance of the senor is different due to the different elastic behaviors of the movable electrode plate of the sensor under the transverse and normal loads. Therefore, such a difference in the input–output analytical relationship makes the existing theory for numerically designing and calibrating the circular non-touch mode capacitive pressure sensors used for measuring the uniformly distributed transverse loads inapplicable to the measurement of the uniformly distributed normal loads.

The improved analytical solution for the large deflection problem of circular membranes under uniformly distributed normal loads in Section 2.1 in this paper is suitable for applications with larger membrane deflections, in comparison with the two existing analytical solutions. 

The input–output analytical relationship and the improved analytical solution, which are derived in Section 2 in this paper, can well meet the needs of the numerical design and calibration of the circular non-touch mode capacitive pressure sensors used for measuring static gas pressure (belonging to uniformly distributed normal loads). 

The results of the investigation into the influence of changing the design parameters of the sensor on its capacitance–pressure (*C*–*q*) analytical relationship in Section 3.5 in this paper have demonstrated clearly the adjusting directions of changing the design parameters to increase or decrease the range of the capacitance and/or pressure, and the degree of influence of changing the design parameters on the *C*–*q* analytical relationship.

The theoretical study presented here provides theoretical support, technical guidance and demonstration, and convenience for application for the numerical design and calibration of circular non-touch mode capacitive pressure sensors used for measuring static gas pressure.

## Figures and Tables

**Table 1:** The analytical expressions of “Function 1” and “Function 2” in Figure 7, and the variation ranges of the output pressure *q* and input capacitance *C*.

Functions	*C*/pF	*q*/KPa	Analytical Expressions
Function 1	2.724~5.499	0~8	*q* = 3.335 × 10^−3^*C* − 1.034 × 10^−2^
Function 2	2.724~18.254	0~16.25	*q* = 1.461 × 10^−7^*C*^4^ + 2.905 × 10^−6^*C*^3^ − 3.111 × 10^−4^*C*^2^ + 5.471 × 10^−3^*C* − 1.371 × 10^−2^

## Data Availability

Data are contained within the article.

## Appendix A

**Table A1:** The numerical calculation results of the undetermined constants *b*_0_ and *d*_0_, maximum membrane deflection *w*_m_, and capacitance *C* when the uniformly distributed normal load *q* takes different values, and *a* = 70 mm, *h* = 0.3 mm, *t* = 0.1 mm, *E* = 3.05 MPa, *ν* = 0.45, and *g* = 50 mm.

*q*/KPa	*b*_0_	*d*_0_	*w*_m_/mm	*C*/pF
0	0	0	0	2.724
0.5	0.056631	0.205102	14.357	3.250
1	0.095145	0.256978	17.988	3.447
2	0.164561	0.320076	22.405	3.751
4	0.294107	0.398152	27.871	4.274
6	0.418627	0.456177	31.932	4.826
8	0.540343	0.507193	35.504	5.499
10	0.660101	0.555241	38.867	6.397
12	0.778356	0.602013	42.141	7.726
14	0.895397	0.648337	45.384	10.037
15	0.953526	0.671478	47.003	12.128
16	1.011422	0.694655	48.626	16.274
16.25	1.025862	0.700458	49.032	18.254

**Table A2:** The numerical calculation results of the undetermined constants *b*_0_ and *d*_0_, maximum membrane deflection *w*_m_, and capacitance *C* when the uniformly distributed transverse load *q* takes different values, and *a* = 70 mm, *h* = 0.3 mm, *t* = 0.1 mm, *E* = 3.05 MPa, *ν* = 0.45, and *g* = 50 mm.

*q*/KPa	*b*_0_	*d*_0_	*w*_m_/mm	*C*/pF
0	0	0	0	2.724
0.5	0.053005	0.207083	14.496	3.244
1	0.085777	0.261467	18.303	3.437
2	0.140470	0.329881	23.092	3.735
4	0.234363	0.414664	29.026	4.237
6	0.319751	0.472816	33.097	4.719
8	0.400731	0.518867	36.321	5.232
10	0.478794	0.558255	39.078	5.822
12	0.554696	0.593610	41.553	6.547
14	0.628895	0.626391	43.847	7.511
15	0.665456	0.642107	44.947	8.144
16	0.701700	0.657484	46.024	8.946
16.25	0.710715	0.661283	46.290	9.183
18	0.773337	0.687467	48.123	11.642
19	0.808772	0.702171	49.152	14.717
19.5	0.826403	0.709468	49.663	18.254

**Table A3:** The numerical calculation results of the undetermined constants *b*_0_ and *d*_0_, maximum membrane deflection *w*_m_, maximum membrane stress *σ*_m_, and capacitance *C* when the uniformly distributed normal load *q* takes different values, and *a* = 70 mm, *h* = 0.3 mm, *t* = 0.1 mm, *E* = 3.05 MPa, *ν* = 0.45, and *g* = 50 mm.

*q*/KPa	*b*_0_	*d*_0_	*w*_m_/mm	*σ*_m_/MPa	*C*/pF
0	0	0	0	0	2.724
0.5	0.056631	0.205102	14.357	0.173	3.250
1	0.095145	0.256978	17.988	0.290	3.447
2	0.164561	0.320076	22.405	0.502	3.751
4	0.294107	0.398152	27.871	0.897	4.274
6	0.418627	0.456177	31.932	1.277	4.826
8	0.540343	0.507193	35.504	1.648	5.499
10	0.660101	0.555241	38.867	2.013	6.397
12	0.778356	0.602013	42.141	2.374	7.726
14	0.895397	0.648337	45.384	2.731	10.037
15	0.953526	0.671478	47.003	2.908	12.128
16	1.011422	0.694655	48.626	3.085	16.274
16.25	1.025862	0.700458	49.032	3.129	18.254

**Table A4:** The numerical calculation results of the undetermined constants *b*_0_ and *d*_0_, maximum membrane deflection *w*_m_, maximum membrane stress *σ*_m_, and capacitance *C* when the uniformly distributed normal load *q* takes different values, and *a* = 70 mm, *h* = 0.6 mm, *t* = 0.1 mm, *E* = 3.05 MPa, *ν* = 0.45, and *g* = 50 mm.

*q*/KPa	*b*_0_	*d*_0_	*w*_m_/mm	*σ*_m_/MPa	*C*/pF
0	0	0	0	0	2.724
0.5	0.034407	0.163158	11.421	0.105	3.116
1	0.056631	0.205102	14.357	0.173	3.250
2	0.095145	0.256978	17.988	0.290	3.447
4	0.164561	0.320076	22.405	0.502	3.751
6	0.230241	0.363350	25.434	0.702	4.016
8	0.294107	0.398152	27.871	0.897	4.274
10	0.356799	0.428481	29.994	1.088	4.540
12	0.418627	0.456177	31.932	1.277	4.826
14	0.479769	0.482222	33.756	1.463	5.142
16	0.540343	0.507193	35.504	1.648	5.499
18	0.600433	0.531453	37.202	1.831	5.911
20	0.660101	0.555241	38.867	2.013	6.397
22	0.719396	0.578722	40.511	2.194	6.987
24	0.778356	0.602013	42.141	2.374	7.726
26	0.837014	0.625197	43.764	2.553	8.691
28	0.895397	0.648337	45.384	2.731	10.037
30	0.953526	0.671478	47.003	2.908	12.128
32.5	1.025862	0.700458	49.032	3.129	18.254

**Table A5:** The numerical calculation results of the undetermined constants *b*_0_ and *d*_0_, maximum membrane deflection *w*_m_, maximum membrane stress *σ*_m_, and capacitance *C* when the uniformly distributed normal load *q* takes different values, and *a* = 70 mm, *h* = 0.9 mm, *t* = 0.1 mm, *E* = 3.05 MPa, *ν* = 0.45, and *g* = 50 mm.

*q*/KPa	*b*_0_	*d*_0_	*w*_m_/mm	*σ*_m_/MPa	*C*/pF
0	0	0	0	0	2.724
0.5	0.025876	0.142622	9.984	0.079	3.056
1	0.042229	0.179453	12.562	0.129	3.166
2	0.070029	0.225352	15.775	0.214	3.323
4	0.118969	0.281714	19.720	0.363	3.557
6	0.164561	0.320076	22.405	0.502	3.751
8	0.208600	0.350179	24.513	0.636	3.929
10	0.251688	0.375624	26.294	0.768	4.101
12	0.294107	0.398152	27.871	0.897	4.274
14	0.336009	0.418730	29.311	1.025	4.450
16	0.377494	0.437945	30.656	1.151	4.633
18	0.418627	0.456177	31.932	1.277	4.826
20	0.459456	0.473683	33.158	1.401	5.033
22	0.500019	0.490644	34.345	1.525	5.256
24	0.540343	0.507193	35.504	1.648	5.499
26	0.580452	0.523429	36.640	1.770	5.766
28	0.620366	0.539426	37.760	1.892	6.064
30	0.660101	0.555241	38.867	2.013	6.397
32	0.699670	0.570922	39.965	2.134	6.777
34	0.739085	0.586502	41.055	2.254	7.214
36	0.778356	0.602013	42.141	2.374	7.726
38	0.817493	0.617477	43.223	2.493	8.337
40	0.856505	0.632913	44.304	2.612	9.086
42	0.895397	0.648337	45.384	2.731	10.037
44	0.934177	0.663762	46.463	2.849	11.306
46	0.972850	0.679198	47.544	2.967	13.145
48	1.011422	0.694655	48.626	3.085	16.274
48.75	1.025862	0.700458	49.032	3.129	18.254

**Table A6:** The numerical calculation results of the undetermined constants *b*_0_ and *d*_0_, maximum membrane deflection *w*_m_, maximum membrane stress *σ*_m_, and capacitance *C* when the uniformly distributed normal load *q* takes different values, and *a* = 70 mm, *h* = 0.3 mm, *t* = 0.1 mm, *E* = 5 MPa, *ν* = 0.45, and *g* = 50 mm.

*q*/KPa	*b*_0_	*d*_0_	*w*_m_/mm	*σ*_m_/MPa	*C*/pF
0	0	0	0	0	2.724
0.5	0.039630	0.174260	12.198	0.198	3.149
1	0.065557	0.218910	15.324	0.328	3.299
2	0.110973	0.273866	19.171	0.555	3.521
4	0.193753	0.340591	23.841	0.969	3.870
6	0.272546	0.386955	27.087	1.363	4.186
8	0.349326	0.425006	29.750	1.747	4.507
10	0.424770	0.458844	32.119	2.124	4.856
12	0.499210	0.490309	34.322	2.496	5.251
14	0.572847	0.520364	36.426	2.864	5.713
16	0.645816	0.549565	38.470	3.229	6.273
18	0.718213	0.578255	40.478	3.591	6.974
20	0.790111	0.606656	42.466	3.951	7.897
22	0.861567	0.634919	44.444	4.308	9.197
24	0.932628	0.663145	46.420	4.663	11.247
26	1.003330	0.691408	48.399	5.017	15.429
26.64	1.025885	0.700469	49.032	5.129	18.258

**Table A7:** The numerical calculation results of the undetermined constants *b*_0_ and *d*_0_, maximum membrane deflection *w*_m_, maximum membrane stress *σ*_m_, and capacitance *C* when the uniformly distributed normal load *q* takes different values, and *a* = 70 mm, *h* = 0.3 mm, *t* = 0.1 mm, *E* = 8 MPa, *ν* = 0.45, and *g* = 50 mm.

*q*/KPa	*b*_0_	*d*_0_	*w*_m_/mm	*σ*_m_/MPa	*C*/pF
0	0	0	0	0	2.724
0.5	0.028425	0.149126	10.439	0.227	3.075
1	0.046509	0.187590	13.131	0.372	3.192
2	0.077434	0.235420	16.479	0.619	3.361
4	0.132299	0.293948	20.576	1.058	3.615
6	0.183696	0.333795	23.366	1.470	3.829
8	0.233470	0.365243	25.567	1.868	4.029
10	0.282234	0.392046	27.443	2.258	4.225
12	0.330274	0.415992	29.119	2.642	4.425
14	0.377752	0.438062	30.664	3.022	4.634
16	0.424770	0.458844	32.119	3.398	4.856
18	0.471398	0.478715	33.510	3.771	5.096
20	0.517688	0.497929	34.855	4.142	5.359
22	0.563681	0.516663	36.166	4.509	5.651
24	0.609409	0.535047	37.453	4.875	5.978
26	0.654895	0.553174	38.722	5.239	6.351
28	0.700163	0.571117	39.978	5.601	6.782
30	0.745230	0.588930	41.225	5.962	7.289
32	0.790111	0.606656	42.466	6.321	7.897
34	0.834820	0.624329	43.703	6.679	8.649
36	0.879368	0.641975	44.938	7.035	9.614
38	0.923765	0.659616	46.173	7.390	10.923
40	0.968021	0.677269	47.409	7.744	12.868
42	1.012144	0.694946	48.646	8.097	16.357
42.623	1.025863	0.700463	49.032	8.207	18.254

**Table A8:** The numerical calculation results of the undetermined constants *b*_0_ and *d*_0_, maximum membrane deflection *w*_m_, maximum membrane stress *σ*_m_, and capacitance *C* when the uniformly distributed normal load *q* takes different values, and *a* = 70 mm, *h* = 0.3 mm, *t* = 0.1 mm, *E* = 3.05 MPa, *ν* = 0.3, and *g* = 50 mm.

*q*/KPa	*b*_0_	*d*_0_	*w*_m_/mm	*σ*_m_/MPa	*C*/pF
0	0	0	0	0	2.724
0.5	0.054367	0.219491	15.364	0.166	3.305
1	0.091708	0.274939	19.246	0.28	3.530
2	0.159330	0.342788	23.995	0.486	3.887
4	0.285631	0.429649	30.075	0.871	4.554
6	0.406905	0.497583	34.831	1.241	5.354
8	0.525354	0.559767	39.184	1.602	6.496
10	0.641842	0.620140	43.410	1.958	8.442
12	0.756837	0.680283	47.620	2.308	13.272
12.665	0.794968	0.700458	49.032	2.425	18.191

**Table A9:** The numerical calculation results of the undetermined constants *b*_0_ and *d*_0_, maximum membrane deflection *w*_m_, maximum membrane stress *σ*_m_, and capacitance *C* when the uniformly distributed normal load *q* takes different values, and *a* = 70 mm, *h* = 0.3 mm, *t* = 0.1 mm, *E* = 3.05 MPa, *ν* = 0.15, and *g* = 50 mm.

*q*/KPa	*b*_0_	*d*_0_	*w*_m_/mm	*σ*_m_/MPa	*C*/pF
0	0	0	0	0	2.724
0.5	0.052733	0.231534	16.207	0.161	3.353
1	0.089173	0.290117	20.308	0.272	3.605
2	0.155281	0.362592	25.381	0.474	4.018
4	0.278657	0.458860	32.120	0.850	4.859
6	0.396969	0.537816	37.647	1.211	6.025
8	0.512441	0.612666	42.887	1.563	8.110
10	0.625963	0.687206	48.104	1.909	14.428
10.353	0.645847	0.700462	49.032	1.970	18.129

**Table A10:** The numerical calculation results of the undetermined constants *b*_0_ and *d*_0_, maximum membrane deflection *w*_m_, maximum membrane stress *σ*_m_, and capacitance *C* when the uniformly distributed normal load *q* takes different values, and *a* = 70 mm, *h* = 0.3 mm, *t* = 5 mm, *E* = 3.05 MPa, *ν* = 0.45, and *g* = 50 mm.

*q*/KPa	*b*_0_	*d*_0_	*w*_m_/mm	*σ*_m_/MPa	*C*/pF
0	0	0	0	0	2.524
0.5	0.056631	0.205102	14.357	0.173	3.105
1	0.095145	0.256978	17.988	0.290	3.284
2	0.164561	0.320076	22.405	0.502	3.559
4	0.294107	0.398152	27.871	0.897	4.026
6	0.418627	0.456177	31.932	1.277	4.513
8	0.540343	0.507193	35.504	1.648	5.096
10	0.660101	0.555241	38.867	2.013	5.858
12	0.778356	0.602013	42.141	2.374	6.953
14	0.895397	0.648337	45.384	2.731	8.770
16	1.011422	0.694655	48.626	3.085	13.187
16.25	1.025862	0.700458	49.032	3.129	14.457

**Table A11:** The numerical calculation results of the undetermined constants *b*_0_ and *d*_0_, maximum membrane deflection *w*_m_, maximum membrane stress *σ*_m_, and capacitance *C* when the uniformly distributed normal load *q* takes different values, and *a* = 70 mm, *h* = 0.3 mm, *t* = 10 mm, *E* = 3.05 MPa, *ν* = 0.45, and *g* = 50 mm.

*q*/KPa	*b*_0_	*d*_0_	*w*_m_/mm	*σ*_m_/MPa	*C*/pF
0	0	0	0	0	2.524
0.5	0.056631	0.205102	14.357	0.173	2.970
1	0.095145	0.256978	17.988	0.290	3.133
2	0.164561	0.320076	22.405	0.502	3.382
4	0.294107	0.398152	27.871	0.897	3.801
6	0.418627	0.456177	31.932	1.277	4.232
8	0.540343	0.507193	35.504	1.648	4.741
10	0.660101	0.555241	38.867	2.013	5.394
12	0.778356	0.602013	42.141	2.374	6.309
14	0.895397	0.648337	45.384	2.731	7.770
16	1.011422	0.694655	48.626	3.085	11.048
16.25	1.025862	0.700458	49.032	3.129	11.926

**Table A12:** The numerical calculation results of the undetermined constants *b*_0_ and *d*_0_, maximum membrane deflection *w*_m_, maximum membrane stress *σ*_m_, and capacitance *C* when the uniformly distributed normal load *q* takes different values, and *a* = 80 mm, *h* = 0.3 mm, *t* = 0.1 mm, *E* = 3.05 MPa, *ν* = 0.45, and *g* = 50 mm.

*q*/KPa	*b*_0_	*d*_0_	*w*_m_/mm	*σ*_m_/MPa	*C*/pF
0	0	0	0	0	3.558
0.5	0.062468	0.214282	17.143	0.191	4.426
1	0.105474	0.268216	21.457	0.322	4.778
2	0.183578	0.333714	26.697	0.560	5.351
4	0.330051	0.415885	33.271	1.007	6.461
6	0.471071	0.478578	38.286	1.437	7.877
8	0.608981	0.534876	42.790	1.857	10.131
10	0.744703	0.588722	47.098	2.271	15.177
10.911	0.805911	0.612899	49.032	2.458	22.243

**Table A13:** The numerical calculation results of the undetermined constants *b*_0_ and *d*_0_, maximum membrane deflection *w*_m_, maximum membrane stress *σ*_m_, and capacitance *C* when the uniformly distributed normal load *q* takes different values, and *a* = 90 mm, *h* = 0.3 mm, *t* = 0.1 mm, *E* = 3.05 MPa, *ν* = 0.45, and *g* = 50 mm.

*q*/KPa	*b*_0_	*d*_0_	*w*_m_/mm	*σ*_m_/MPa	*C*/pF
0	0	0	0	0	4.503
0.5	0.068159	0.222694	20.042	0.208	5.870
1	0.115620	0.278478	25.063	0.353	6.476
2	0.202376	0.346220	31.160	0.617	7.543
4	0.365680	0.432569	38.931	1.115	10.021
6	0.523089	0.500144	45.013	1.595	14.669
7	0.600433	0.531453	47.831	1.831	20.413
7.435	0.633853	0.544804	49.032	1.933	26.553

**Table A14:** The numerical calculation results of the undetermined constants *b*_0_ and *d*_0_, maximum membrane deflection *w*_m_, maximum membrane stress *σ*_m_, and capacitance *C* when the uniformly distributed normal load *q* takes different values, and *a* = 70 mm, *h* = 0.3 mm, *t* = 0.1 mm, *E* = 3.05 MPa, *ν* = 0.45, and *g* = 55 mm.

*q*/KPa	*b*_0_	*d*_0_	*w*_m_/mm	*σ*_m_/MPa	*C*/pF
0	0	0	0	0	2.477
0.5	0.056631	0.205102	14.357	0.173	2.901
1	0.095145	0.256978	17.988	0.290	3.055
2	0.164561	0.320076	22.405	0.502	3.288
4	0.294107	0.398152	27.871	0.897	3.673
6	0.418627	0.456177	31.932	1.277	4.061
8	0.540343	0.507193	35.504	1.648	4.508
10	0.660101	0.555241	38.867	2.013	5.061
12	0.778356	0.602013	42.141	2.374	5.790
14	0.895397	0.648337	45.384	2.731	6.823
16	1.011422	0.694655	48.626	3.085	8.466
16.25	1.025862	0.700458	49.032	3.129	8.748

**Table A15:** The numerical calculation results of the undetermined constants *b*_0_ and *d*_0_, maximum membrane deflection *w*_m_, maximum membrane stress *σ*_m_, and capacitance *C* when the uniformly distributed normal load *q* takes different values, and *a* = 70 mm, *h* = 0.3 mm, *t* = 0.1 mm, *E* = 3.05 MPa, *ν* = 0.45, and *g* = 60 mm.

*q*/KPa	*b*_0_	*d*_0_	*w*_m_/mm	*σ*_m_/MPa	*C*/pF
0	0	0	0	0	2.270
0.5	0.056631	0.205102	14.357	0.173	2.621
1	0.095145	0.256978	17.988	0.290	2.744
2	0.164561	0.320076	22.405	0.502	2.928
4	0.294107	0.398152	27.871	0.897	3.224
6	0.418627	0.456177	31.932	1.277	3.513
8	0.540343	0.507193	35.504	1.648	3.833
10	0.660101	0.555241	38.867	2.013	4.212
12	0.778356	0.602013	42.141	2.374	4.681
14	0.895397	0.648337	45.384	2.731	5.290
16	1.011422	0.694655	48.626	3.085	6.127
16.25	1.025862	0.700458	49.032	3.129	6.255

## Appendix B

The following are the recursion formulas for the power series coefficients *b_i_*, *c_i_*, and *d_i_* in Equations (28), (29) and (30), where *Q* is a dimensionless variable (see Equation (21)), *v* is the Poisson’s ratio, the coefficients *b_i_*, *c_i_*, and *d_i_* are constantly equal to zero when *i* = 1, 3, 5,…, and *c*_0_ ≡ *b*_0_.

$$b_{2}=-\frac{Q^{2}\left(4v^{2}b_{0}^{2}+8vb_{0}^{2}-4vb_{0}-12b_{0}^{2}-12b_{0}-1\right)}{64b_{0}^{2}\left(vb_{0}-b_{0}-1\right)},$$

$$\begin{array}{l} b_{4}=\frac{1}{96b_{0}^{2}\left(vb_{0}-b_{0}-1\right)}(Q^{3}v^{2}b_{0}d_{2}-8Q\;v^{2}b_{0}^{2}b_{2}d_{2}+4Q^{3}vb_{0}d_{2}-32Qvb_{0}^{2}b_{2}d_{2}+18v^{2}b_{0}^{2}b_{2}^{2}\\ -2v^{2}b_{0}^{2}c_{2}^{2}-Q^{3}vd_{2}-5Q^{3}b_{0}d_{2}+8Qvb_{0}b_{2}d_{2}+40Qb_{0}^{2}b_{2}d_{2}-32vb_{0}^{2}b_{2}c_{2}-5Q^{3}d_{2}+2Q^{2}d_{2}^{2}\\ +40Qb_{0}b_{2}d_{2}-2b_{0}^{2}b_{2}^{2}+18b_{0}^{2}c_{2}^{2}-16b_{0}b_{2}d_{2}^{2}) \end{array},$$

$$\begin{array}{l} b_{6}=\frac{1}{48b_{0}^{2}d_{2}\left(vb_{0}-b_{0}-1\right)}(Q^{3}v^{2}b_{0}d_{2}d_{4}-4Q\;v^{2}b_{0}^{3}d_{4}^{2}-8Q\;v^{2}b_{0}^{2}b_{2}d_{2}d_{4}-2Q\;v^{2}b_{0}^{2}b_{4}d_{2}^{2}-Q\;v^{2}b_{0}b_{2}^{2}d_{2}^{2}\\ +28Qb_{0}^{3}d_{4}^{2}+56Qb_{0}^{2}b_{2}d_{2}d_{4}+14Qb_{0}^{2}b_{4}d_{2}^{2}+7Qb_{0}b_{2}^{2}d_{2}^{2}-14vb_{0}^{2}b_{2}c_{4}d_{2}-14vb_{0}^{2}b_{4}c_{2}d_{2}-7Q^{3}d_{2}d_{4}\\ -v^{2}b_{0}^{2}c_{2}c_{4}d_{2}-Q^{3}vd_{2}d_{4}-7Q^{3}b_{0}d_{2}d_{4}+4Qvb_{0}^{2}d_{4}^{2}+8Qvb_{0}b_{2}d_{2}d_{4}+2Qvb_{0}b_{4}d_{2}^{2}+Qvb_{2}^{2}d_{2}^{2}\\ +6Q^{3}vb_{0}d_{2}d_{4}-24Qvb_{0}^{3}d_{4}^{2}-48Qvb_{0}^{2}b_{2}d_{2}d_{4}-12Qvb_{0}^{2}b_{4}d_{2}^{2}-6Qvb_{0}b_{2}^{2}d_{2}^{2}+15v^{2}b_{0}^{2}b_{2}b_{4}d_{2}\\ +2Q^{2}d_{2}^{2}d_{4}+28Qb_{0}^{2}d_{4}^{2}+56Qb_{0}b_{2}d_{2}d_{4}+14Qb_{0}b_{4}d_{2}^{2}+7Qb_{2}^{2}d_{2}^{2}-b_{0}^{2}b_{2}b_{4}d_{2}+15b_{0}^{2}c_{2}c_{4}d_{2}\\ -16b_{0}b_{2}d_{2}^{2}d_{4}-4b_{0}b_{4}d_{2}^{3}-2b_{2}^{2}d_{2}^{3}) \end{array},$$

$$\begin{array}{l} b_{8}=\frac{1}{160b_{0}^{2}d_{2}\left(vb_{0}-b_{0}-1\right)}(3Q^{3}v^{2}b_{0}d_{2}d_{6}+2Q^{3}v^{2}b_{0}d_{4}^{2}-24Q\;v^{2}b_{0}^{3}d_{4}d_{6}-24Q\;v^{2}b_{0}^{2}b_{2}d_{2}d_{6}\\ +25v^{2}b_{0}^{2}b_{4}^{2}d_{2}-2v^{2}b_{0}^{2}c_{2}c_{6}d_{2}-v^{2}b_{0}^{2}c_{4}^{2}d_{2}-3Q^{3}vd_{2}d_{6}-2Q^{3}vd_{4}^{2}-27Q^{3}b_{0}d_{2}d_{6}-18Q^{3}b_{0}d_{4}^{2}\\ +72Qb_{2}^{2}d_{2}d_{4}+36Qb_{2}b_{4}d_{2}^{2}-2b_{0}^{2}b_{2}b_{6}d_{2}-b_{0}^{2}b_{4}^{2}d_{2}+42b_{0}^{2}c_{2}c_{6}d_{2}+25b_{0}^{2}c_{4}^{2}d_{2}-48b_{0}b_{2}d_{2}^{2}d_{6}\\ -128Qvb_{0}^{2}b_{4}d_{2}d_{4}-32Qvb_{0}^{2}b_{6}d_{2}^{2}-64Qvb_{0}b_{2}^{2}d_{2}d_{4}-32Qvb_{0}b_{2}b_{4}d_{2}^{2}+42v^{2}b_{0}^{2}b_{2}b_{6}d_{2}\\ -16Q\;v^{2}b_{0}^{2}b_{2}d_{4}^{2}-16Q\;v^{2}b_{0}^{2}b_{4}d_{2}d_{4}-4Q\;v^{2}b_{0}^{2}b_{6}d_{2}^{2}-8Q\;v^{2}b_{0}b_{2}^{2}d_{2}d_{4}-4Q\;v^{2}b_{0}b_{2}b_{4}d_{2}^{2}\\ +24Q^{3}vb_{0}d_{2}d_{6}+16Q^{3}vb_{0}d_{4}^{2}-192Qvb_{0}^{3}d_{4}d_{6}-192Qvb_{0}^{2}b_{2}d_{2}d_{6}-128Qvb_{0}^{2}b_{2}d_{4}^{2}\\ +216Qb_{0}^{2}d_{4}d_{6}+216Qb_{0}b_{2}d_{2}d_{6}+144Qb_{0}b_{2}d_{4}^{2}+144Qb_{0}b_{4}d_{2}d_{4}+36Qb_{0}b_{6}d_{2}^{2}\\ +24Qvb_{0}^{2}d_{4}d_{6}+24Qvb_{0}b_{2}d_{2}d_{6}+16Qvb_{0}b_{2}d_{4}^{2}+16Qvb_{0}b_{4}d_{2}d_{4}+4Qvb_{0}b_{6}d_{2}^{2}\\ +144Qb_{0}^{2}b_{4}d_{2}d_{4}+36Qb_{0}^{2}b_{6}d_{2}^{2}+72Qb_{0}b_{2}^{2}d_{2}d_{4}+36Qb_{0}b_{2}b_{4}d_{2}^{2}-40vb_{0}^{2}b_{2}c_{6}d_{2}\\ -48vb_{0}^{2}b_{4}c_{4}d_{2}-40vb_{0}^{2}b_{6}c_{2}d_{2}-27Q^{3}d_{2}d_{6}-18Q^{3}d_{4}^{2}+6Q^{2}d_{2}^{2}d_{6}+4Q^{2}d_{2}d_{4}^{2}\\ +8Qvb_{2}^{2}d_{2}d_{4}+4Qvb_{2}b_{4}d_{2}^{2}+216Qb_{0}^{3}d_{4}d_{6}+216Qb_{0}^{2}b_{2}d_{2}d_{6}+144Qb_{0}^{2}b_{2}d_{4}^{2}\\ -32b_{0}b_{2}d_{2}d_{4}^{2}-32b_{0}b_{4}d_{2}^{2}d_{4}-8b_{0}b_{6}d_{2}^{3}-16b_{2}^{2}d_{2}^{2}d_{4}-8b_{2}b_{4}d_{2}^{3}) \end{array},$$

$$\begin{array}{l} b_{10}=\frac{1}{120b_{0}^{2}d_{2}\left(vb_{0}-b_{0}-1\right)}(2Q^{3}v^{2}b_{0}d_{2}d_{8}+3Q^{3}v^{2}b_{0}d_{4}d_{6}-16Q\;v^{2}b_{0}^{3}d_{4}d_{8}-9Q\;v^{2}b_{0}^{3}d_{6}^{2}\\ -160Qvb_{0}^{2}b_{2}d_{2}d_{8}-240Qvb_{0}^{2}b_{2}d_{4}d_{6}-120Qvb_{0}^{2}b_{4}d_{2}d_{6}-80Qvb_{0}^{2}b_{4}d_{4}^{2}-80Qvb_{0}^{2}b_{6}d_{2}d_{4}\\ -16Q\;v^{2}b_{0}^{2}b_{2}d_{2}d_{8}-24Q\;v^{2}b_{0}^{2}b_{2}d_{4}d_{6}-12Q\;v^{2}b_{0}^{2}b_{4}d_{2}d_{6}-8Q\;v^{2}b_{0}^{2}b_{4}d_{4}^{2}-8Q\;v^{2}b_{0}^{2}b_{6}d_{2}d_{4}\\ -10Qvb_{0}b_{4}^{2}d_{2}^{2}+27v^{2}b_{0}^{2}b_{2}b_{8}d_{2}+35v^{2}b_{0}^{2}b_{4}b_{6}d_{2}-v^{2}b_{0}^{2}c_{2}c_{8}d_{2}-v^{2}b_{0}^{2}c_{4}c_{6}d_{2}-2Q^{3}vd_{2}d_{8}\\ -3Q^{3}vd_{4}d_{6}-22Q^{3}b_{0}d_{2}d_{8}-33Q^{3}b_{0}d_{4}d_{6}+16Qvb_{0}^{2}d_{4}d_{8}+9Qvb_{0}^{2}d_{6}^{2}+16Qvb_{0}b_{2}d_{2}d_{8}\\ +88Qb_{0}b_{6}d_{2}d_{4}+22Qb_{0}b_{8}d_{2}^{2}+66Qb_{2}^{2}d_{2}d_{6}+44Qb_{2}^{2}d_{4}^{2}+88Qb_{2}b_{4}d_{2}d_{4}+22Qb_{2}b_{6}d_{2}^{2}\\ -20Qvb_{0}^{2}b_{8}d_{2}^{2}-60Qvb_{0}b_{2}^{2}d_{2}d_{6}-40Qvb_{0}b_{2}^{2}d_{4}^{2}-80Qvb_{0}b_{2}b_{4}d_{2}d_{4}-20Qvb_{0}b_{2}b_{6}d_{2}^{2}\\ -2Q\;v^{2}b_{0}^{2}b_{8}d_{2}^{2}-6Q\;v^{2}b_{0}b_{2}^{2}d_{2}d_{6}-4Q\;v^{2}b_{0}b_{2}^{2}d_{4}^{2}-8Q\;v^{2}b_{0}b_{2}b_{4}d_{2}d_{4}-2Q\;v^{2}b_{0}b_{2}b_{6}d_{2}^{2}\\ -26vb_{0}^{2}b_{8}c_{2}d_{2}-22Q^{3}d_{2}d_{8}-33Q^{3}d_{4}d_{6}+4Q^{2}d_{2}^{2}d_{8}+6Q^{2}d_{2}d_{4}d_{6}+176Qb_{0}^{2}d_{4}d_{8}\\ +6Qvb_{2}^{2}d_{2}d_{6}+4Qvb_{2}^{2}d_{4}^{2}+8Qvb_{2}b_{4}d_{2}d_{4}+2Qvb_{2}b_{6}d_{2}^{2}+Qvb_{4}^{2}d_{2}^{2}+176Qb_{0}^{3}d_{4}d_{8}\\ -48b_{0}b_{2}d_{2}d_{4}d_{6}-24b_{0}b_{4}d_{2}^{2}d_{6}-16b_{0}b_{4}d_{2}d_{4}^{2}-16b_{0}b_{6}d_{2}^{2}d_{4}-4b_{0}b_{8}d_{2}^{3}-12b_{2}^{2}d_{2}^{2}d_{6}\\ +24Qvb_{0}b_{2}d_{4}d_{6}+12Qvb_{0}b_{4}d_{2}d_{6}+8Qvb_{0}b_{4}d_{4}^{2}+8Qvb_{0}b_{6}d_{2}d_{4}+2Qvb_{0}b_{8}d_{2}^{2}\\ +99Qb_{0}^{3}d_{6}^{2}+176Qb_{0}^{2}b_{2}d_{2}d_{8}+264Qb_{0}^{2}b_{2}d_{4}d_{6}+132Qb_{0}^{2}b_{4}d_{2}d_{6}+88Qb_{0}^{2}b_{4}d_{4}^{2}\\ +88Qb_{0}^{2}b_{6}d_{2}d_{4}+22Qb_{0}^{2}b_{8}d_{2}^{2}+66Qb_{0}b_{2}^{2}d_{2}d_{6}+44Qb_{0}b_{2}^{2}d_{4}^{2}+88Qb_{0}b_{2}b_{4}d_{2}d_{4}\\ +99Qb_{0}^{2}d_{6}^{2}+176Qb_{0}b_{2}d_{2}d_{8}+264Qb_{0}b_{2}d_{4}d_{6}+132Qb_{0}b_{4}d_{2}d_{6}+88Qb_{0}b_{4}d_{4}^{2}\\ +11Qb_{4}^{2}d_{2}^{2}-b_{0}^{2}b_{2}b_{8}d_{2}-b_{0}^{2}b_{4}b_{6}d_{2}+27b_{0}^{2}c_{2}c_{8}d_{2}+35b_{0}^{2}c_{4}c_{6}d_{2}-32b_{0}b_{2}d_{2}^{2}d_{8}\\ -Q\;v^{2}b_{0}b_{4}^{2}d_{2}^{2}+20Q^{3}vb_{0}d_{2}d_{8}+30Q^{3}vb_{0}d_{4}d_{6}-160Qvb_{0}^{3}d_{4}d_{8}-90Qvb_{0}^{3}d_{6}^{2}\\ +22Qb_{0}b_{2}b_{6}d_{2}^{2}+11Qb_{0}b_{4}^{2}d_{2}^{2}-26vb_{0}^{2}b_{2}c_{8}d_{2}-34vb_{0}^{2}b_{4}c_{6}d_{2}-34vb_{0}^{2}b_{6}c_{4}d_{2}\\ -8b_{2}^{2}d_{2}d_{4}^{2}-16b_{2}b_{4}d_{2}^{2}d_{4}-4b_{2}b_{6}d_{2}^{3}-2b_{4}^{2}d_{2}^{3}) \end{array},$$

$$\begin{array}{l} b_{12}=\frac{1}{672b_{0}^{2}d_{2}\left(vb_{0}-b_{0}-1\right)}(10Q^{3}v^{2}b_{0}d_{2}d_{10}+16Q^{3}v^{2}b_{0}d_{4}d_{8}+9Q^{3}v^{2}b_{0}d_{6}^{2}-80Q\;v^{2}b_{0}^{3}d_{4}d_{10}\\ -1536Qvb_{0}^{2}b_{2}d_{4}d_{8}-864Qvb_{0}^{2}b_{2}d_{6}^{2}-768Qvb_{0}^{2}b_{4}d_{2}d_{8}-1152Qvb_{0}^{2}b_{4}d_{4}d_{6}-576Qvb_{0}^{2}b_{6}d_{2}d_{6}\\ -32Q\;v^{2}b_{0}b_{2}b_{6}d_{2}d_{4}-8Q\;v^{2}b_{0}b_{2}b_{8}d_{2}^{2}-16Q\;v^{2}b_{0}b_{4}^{2}d_{2}d_{4}-8Q\;v^{2}b_{0}b_{4}b_{6}d_{2}^{2}+120Q^{3}vb_{0}d_{2}d_{10}\\ -96Q\;v^{2}b_{0}^{3}d_{6}d_{8}-80Q\;v^{2}b_{0}^{2}b_{2}d_{2}d_{10}-128Q\;v^{2}b_{0}^{2}b_{2}d_{4}d_{8}-72Q\;v^{2}b_{0}^{2}b_{2}d_{6}^{2}-64Q\;v^{2}b_{0}^{2}b_{4}d_{2}d_{8}\\ -384Qvb_{0}^{2}b_{6}d_{4}^{2}-384Qvb_{0}^{2}b_{8}d_{2}d_{4}-96Qvb_{0}^{2}b_{10}d_{2}^{2}-384Qvb_{0}b_{2}^{2}d_{2}d_{8}-576Qvb_{0}b_{2}^{2}d_{4}d_{6}\\ -128b_{0}b_{4}d_{2}^{2}d_{8}-192b_{0}b_{4}d_{2}d_{4}d_{6}-96b_{0}b_{6}d_{2}^{2}d_{6}-64b_{0}b_{6}d_{2}d_{4}^{2}-64b_{0}b_{8}d_{2}^{2}d_{4}-16b_{0}b_{10}d_{2}^{3}\\ -176vb_{0}^{2}b_{8}c_{4}d_{2}-128vb_{0}^{2}b_{10}c_{2}d_{2}-130Q^{3}d_{2}d_{10}-208Q^{3}d_{4}d_{8}-117Q^{3}d_{6}^{2}+20Q^{2}d_{2}^{2}d_{10}\\ +1664Qb_{0}^{2}b_{2}d_{4}d_{8}+936Qb_{0}^{2}b_{2}d_{6}^{2}+832Qb_{0}^{2}b_{4}d_{2}d_{8}+1248Qb_{0}^{2}b_{4}d_{4}d_{6}+624Qb_{0}^{2}b_{6}d_{2}d_{6}\\ -96Q\;v^{2}b_{0}^{2}b_{4}d_{4}d_{6}-48Q\;v^{2}b_{0}^{2}b_{6}d_{2}d_{6}-32Q\;v^{2}b_{0}^{2}b_{6}d_{4}^{2}-32Q\;v^{2}b_{0}^{2}b_{8}d_{2}d_{4}-8Q\;v^{2}b_{0}^{2}b_{10}d_{2}^{2}\\ +192Q^{3}vb_{0}d_{4}d_{8}+108Q^{3}vb_{0}d_{6}^{2}-960Qvb_{0}^{3}d_{4}d_{10}-1152Qvb_{0}^{3}d_{6}d_{8}-960Qvb_{0}^{2}b_{2}d_{2}d_{10}\\ +80Qvb_{0}b_{2}d_{2}d_{10}+128Qvb_{0}b_{2}d_{4}d_{8}+72Qvb_{0}b_{2}d_{6}^{2}+64Qvb_{0}b_{4}d_{2}d_{8}+96Qvb_{0}b_{4}d_{4}d_{6}\\ -192Qvb_{0}b_{4}^{2}d_{2}d_{4}-96Qvb_{0}b_{4}b_{6}d_{2}^{2}+132v^{2}b_{0}^{2}b_{2}b_{10}d_{2}+180v^{2}b_{0}^{2}b_{4}b_{8}d_{2}+98v^{2}b_{0}^{2}b_{6}^{2}d_{2}\\ +208Qb_{0}b_{4}^{2}d_{2}d_{4}+104Qb_{0}b_{4}b_{6}d_{2}^{2}-128vb_{0}^{2}b_{2}c_{10}d_{2}-176vb_{0}^{2}b_{4}c_{8}d_{2}-192vb_{0}^{2}b_{6}c_{6}d_{2}\\ +416Qb_{0}^{2}b_{6}d_{4}^{2}+416Qb_{0}^{2}b_{8}d_{2}d_{4}+104Qb_{0}^{2}b_{10}d_{2}^{2}+416Qb_{0}b_{2}^{2}d_{2}d_{8}+624Qb_{0}b_{2}^{2}d_{4}d_{6}\\ +208Qb_{4}^{2}d_{2}d_{4}+104Qb_{4}b_{6}d_{2}^{2}-4b_{0}^{2}b_{2}b_{10}d_{2}-4b_{0}^{2}b_{4}b_{8}d_{2}-2b_{0}^{2}b_{6}^{2}d_{2}+132b_{0}^{2}c_{2}c_{10}d_{2}\\ +16Qvb_{4}^{2}d_{2}d_{4}+8Qvb_{4}b_{6}d_{2}^{2}+1040Qb_{0}^{3}d_{4}d_{10}+1248Qb_{0}^{3}d_{6}d_{8}+1040Qb_{0}^{2}b_{2}d_{2}d_{10}\\ -4v^{2}b_{0}^{2}c_{2}c_{10}d_{2}-4v^{2}b_{0}^{2}c_{4}c_{8}d_{2}-2v^{2}b_{0}^{2}c_{6}^{2}d_{2}-10Q^{3}vd_{2}d_{10}-16Q^{3}vd_{4}d_{8}-9Q^{3}vd_{6}^{2}\\ +32Q^{2}d_{2}d_{4}d_{8}+18Q^{2}d_{2}d_{6}^{2}+1040Qb_{0}^{2}d_{4}d_{10}+1248Qb_{0}^{2}d_{6}d_{8}+1040Qb_{0}b_{2}d_{2}d_{10}\\ -64b_{2}^{2}d_{2}^{2}d_{8}-96b_{2}^{2}d_{2}d_{4}d_{6}-96b_{2}b_{4}d_{2}^{2}d_{6}-64b_{2}b_{4}d_{2}d_{4}^{2}-64b_{2}b_{6}d_{2}^{2}d_{4}-16b_{2}b_{8}d_{2}^{3}\\ +624Qb_{0}b_{6}d_{2}d_{6}+416Qb_{0}b_{6}d_{4}^{2}+416Qb_{0}b_{8}d_{2}d_{4}+104Qb_{0}b_{10}d_{2}^{2}+416Qb_{2}^{2}d_{2}d_{8}\\ +624Qb_{2}^{2}d_{4}d_{6}+624Qb_{2}b_{4}d_{2}d_{6}+416Qb_{2}b_{4}d_{4}^{2}+416Qb_{2}b_{6}d_{2}d_{4}+104Qb_{2}b_{8}d_{2}^{2}\\ +48Qvb_{0}b_{6}d_{2}d_{6}+32Qvb_{0}b_{6}d_{4}^{2}+32Qvb_{0}b_{8}d_{2}d_{4}+8Qvb_{0}b_{10}d_{2}^{2}+32Qvb_{2}^{2}d_{2}d_{8}\\ +48Qvb_{2}^{2}d_{4}d_{6}+48Qvb_{2}b_{4}d_{2}d_{6}+32Qvb_{2}b_{4}d_{4}^{2}+32Qvb_{2}b_{6}d_{2}d_{4}+8Qvb_{2}b_{8}d_{2}^{2}\\ -130Q^{3}b_{0}d_{2}d_{10}-208Q^{3}b_{0}d_{4}d_{8}-117Q^{3}b_{0}d_{6}^{2}+80Qvb_{0}^{2}d_{4}d_{10}+96Qvb_{0}^{2}d_{6}d_{8}\\ -576Qvb_{0}b_{2}b_{4}d_{2}d_{6}-384Qvb_{0}b_{2}b_{4}d_{4}^{2}-384Qvb_{0}b_{2}b_{6}d_{2}d_{4}-96Qvb_{0}b_{2}b_{8}d_{2}^{2}\\ +180b_{0}^{2}c_{4}c_{8}d_{2}+98b_{0}^{2}c_{6}^{2}d_{2}-160b_{0}b_{2}d_{2}^{2}d_{10}-256b_{0}b_{2}d_{2}d_{4}d_{8}-144b_{0}b_{2}d_{2}d_{6}^{2}\\ -32Q\;v^{2}b_{0}b_{2}^{2}d_{2}d_{8}-48Q\;v^{2}b_{0}b_{2}^{2}d_{4}d_{6}-48Q\;v^{2}b_{0}b_{2}b_{4}d_{2}d_{6}-32Q\;v^{2}b_{0}b_{2}b_{4}d_{4}^{2}\\ +624Qb_{0}b_{2}b_{4}d_{2}d_{6}+416Qb_{0}b_{2}b_{4}d_{4}^{2}+416Qb_{0}b_{2}b_{6}d_{2}d_{4}+104Qb_{0}b_{2}b_{8}d_{2}^{2}\\ +1664Qb_{0}b_{2}d_{4}d_{8}+936Qb_{0}b_{2}d_{6}^{2}+832Qb_{0}b_{4}d_{2}d_{8}+1248Qb_{0}b_{4}d_{4}d_{6}\\ -32b_{4}^{2}d_{2}^{2}d_{4}-16b_{4}b_{6}d_{2}^{3}) \end{array},$$

$$c_{2}=-\frac{Q^{2}\left(12v^{2}b_{0}^{2}-8vb_{0}^{2}-12vb_{0}-4b_{0}^{2}-4b_{0}-3\right)}{64b_{0}^{2}\left(vb_{0}-b_{0}-1\right)},$$

$$\begin{array}{l} c_{4}=\frac{1}{96b_{0}^{2}\left(vb_{0}-b_{0}-1\right)}(5Q^{3}v^{2}b_{0}d_{2}-40Q\;v^{2}b_{0}^{2}b_{2}d_{2}-4Q^{3}vb_{0}d_{2}+32Qvb_{0}^{2}b_{2}d_{2}+90v^{2}b_{0}^{2}b_{2}^{2}\\ -10v^{2}b_{0}^{2}c_{2}^{2}-5Q^{3}vd_{2}-Q^{3}b_{0}d_{2}+40Qvb_{0}b_{2}d_{2}+8Qb_{0}^{2}b_{2}d_{2}-160vb_{0}^{2}b_{2}c_{2}-Q^{3}d_{2}+10Q^{2}d_{2}^{2}\\ +8Qb_{0}b_{2}d_{2}-10b_{0}^{2}b_{2}^{2}+90b_{0}^{2}c_{2}^{2}-80b_{0}b_{2}d_{2}^{2}) \end{array},$$

$$\begin{array}{l} c_{6}=\frac{1}{48b_{0}^{2}d_{2}\left(vb_{0}-b_{0}-1\right)}(7Q^{3}v^{2}b_{0}d_{2}d_{4}-28Q\;v^{2}b_{0}^{3}d_{4}^{2}-56Q\;v^{2}b_{0}^{2}b_{2}d_{2}d_{4}-14Q\;v^{2}b_{0}^{2}b_{4}d_{2}^{2}\\ -7Q\;v^{2}b_{0}b_{2}^{2}d_{2}^{2}-6Q^{3}vb_{0}d_{2}d_{4}+24Qvb_{0}^{3}d_{4}^{2}+48Qvb_{0}^{2}b_{2}d_{2}d_{4}+12Qvb_{0}^{2}b_{4}d_{2}^{2}+6Qvb_{0}b_{2}^{2}d_{2}^{2}\\ +105v^{2}b_{0}^{2}b_{2}b_{4}d_{2}-7v^{2}b_{0}^{2}c_{2}c_{4}d_{2}-7Q^{3}vd_{2}d_{4}-Q^{3}b_{0}d_{2}d_{4}+28Qvb_{0}^{2}d_{4}^{2}+56Qvb_{0}b_{2}d_{2}d_{4}\\ +2Qb_{0}b_{4}d_{2}^{2}+Qb_{2}^{2}d_{2}^{2}-7b_{0}^{2}b_{2}b_{4}d_{2}+105b_{0}^{2}c_{2}c_{4}d_{2}-112b_{0}b_{2}d_{2}^{2}d_{4}-28b_{0}b_{4}d_{2}^{3}-14b_{2}^{2}d_{2}^{3}\\ -98vb_{0}^{2}b_{2}c_{4}d_{2}-98vb_{0}^{2}b_{4}c_{2}d_{2}-Q^{3}d_{2}d_{4}+14Q^{2}d_{2}^{2}d_{4}+4Qb_{0}^{2}d_{4}^{2}+8Qb_{0}b_{2}d_{2}d_{4}\\ +14Qvb_{0}b_{4}d_{2}^{2}+7Qvb_{2}^{2}d_{2}^{2}+4Qb_{0}^{3}d_{4}^{2}+8Qb_{0}^{2}b_{2}d_{2}d_{4}+2Qb_{0}^{2}b_{4}d_{2}^{2}+Qb_{0}b_{2}^{2}d_{2}^{2}) \end{array},$$

$$\begin{array}{l} c_{8}=\frac{1}{160b_{0}^{2}d_{2}\left(vb_{0}-b_{0}-1\right)}(27Q^{3}v^{2}b_{0}d_{2}d_{6}+18Q^{3}v^{2}b_{0}d_{4}^{2}-216Q\;v^{2}b_{0}^{3}d_{4}d_{6}-216Q\;v^{2}b_{0}^{2}b_{2}d_{2}d_{6}\\ +225v^{2}b_{0}^{2}b_{4}^{2}d_{2}-18v^{2}b_{0}^{2}c_{2}c_{6}d_{2}-9v^{2}b_{0}^{2}c_{4}^{2}d_{2}-27Q^{3}vd_{2}d_{6}-18Q^{3}vd_{4}^{2}-3Q^{3}b_{0}d_{2}d_{6}-2Q^{3}b_{0}d_{4}^{2}\\ -24Q^{3}vb_{0}d_{2}d_{6}-16Q^{3}vb_{0}d_{4}^{2}+192Qvb_{0}^{3}d_{4}d_{6}+192Qvb_{0}^{2}b_{2}d_{2}d_{6}+128Qvb_{0}^{2}b_{2}d_{4}^{2}-72b_{2}b_{4}d_{2}^{3}\\ +16Qb_{0}b_{4}d_{2}d_{4}+4Qb_{0}b_{6}d_{2}^{2}+8Qb_{2}^{2}d_{2}d_{4}+4Qb_{2}b_{4}d_{2}^{2}-18b_{0}^{2}b_{2}b_{6}d_{2}-9b_{0}^{2}b_{4}^{2}d_{2}+378b_{0}^{2}c_{2}c_{6}d_{2}\\ +4Qb_{0}^{2}b_{6}d_{2}^{2}+8Qb_{0}b_{2}^{2}d_{2}d_{4}+4Qb_{0}b_{2}b_{4}d_{2}^{2}-360vb_{0}^{2}b_{2}c_{6}d_{2}-432vb_{0}^{2}b_{4}c_{4}d_{2}-360vb_{0}^{2}b_{6}c_{2}d_{2}\\ -3Q^{3}d_{2}d_{6}-2Q^{3}d_{4}^{2}+54Q^{2}d_{2}^{2}d_{6}+36Q^{2}d_{2}d_{4}^{2}+24Qb_{0}^{2}d_{4}d_{6}+24Qb_{0}b_{2}d_{2}d_{6}+16Qb_{0}b_{2}d_{4}^{2}\\ -144Q\;v^{2}b_{0}^{2}b_{2}d_{4}^{2}-144Q\;v^{2}b_{0}^{2}b_{4}d_{2}d_{4}-36Q\;v^{2}b_{0}^{2}b_{6}d_{2}^{2}-72Q\;v^{2}b_{0}b_{2}^{2}d_{2}d_{4}-36Q\;v^{2}b_{0}b_{2}b_{4}d_{2}^{2}\\ +72Qvb_{2}^{2}d_{2}d_{4}+36Qvb_{2}b_{4}d_{2}^{2}+24Qb_{0}^{3}d_{4}d_{6}+24Qb_{0}^{2}b_{2}d_{2}d_{6}+16Qb_{0}^{2}b_{2}d_{4}^{2}+16Qb_{0}^{2}b_{4}d_{2}d_{4}\\ +128Qvb_{0}^{2}b_{4}d_{2}d_{4}+32Qvb_{0}^{2}b_{6}d_{2}^{2}+64Qvb_{0}b_{2}^{2}d_{2}d_{4}+32Qvb_{0}b_{2}b_{4}d_{2}^{2}+378v^{2}b_{0}^{2}b_{2}b_{6}d_{2}\\ +216Qvb_{0}^{2}d_{4}d_{6}+216Qvb_{0}b_{2}d_{2}d_{6}+144Qvb_{0}b_{2}d_{4}^{2}+144Qvb_{0}b_{4}d_{2}d_{4}+36Qvb_{0}b_{6}d_{2}^{2}\\ +225b_{0}^{2}c_{4}^{2}d_{2}-432b_{0}b_{2}d_{2}^{2}d_{6}-288b_{0}b_{2}d_{2}d_{4}^{2}-288b_{0}b_{4}d_{2}^{2}d_{4}-72b_{0}b_{6}d_{2}^{3}-144b_{2}^{2}d_{2}^{2}d_{4}) \end{array},$$

$$\begin{array}{l} c_{10}=\frac{1}{120b_{0}^{2}d_{2}\left(vb_{0}-b_{0}-1\right)}(22Q^{3}v^{2}b_{0}d_{2}d_{8}+33Q^{3}v^{2}b_{0}d_{4}d_{6}-176Q\;v^{2}b_{0}^{3}d_{4}d_{8}-99Q\;v^{2}b_{0}^{3}d_{6}^{2}\\ +385b_{0}^{2}c_{4}c_{6}d_{2}-352b_{0}b_{2}d_{2}^{2}d_{8}-528b_{0}b_{2}d_{2}d_{4}d_{6}-264b_{0}b_{4}d_{2}^{2}d_{6}-176b_{0}b_{4}d_{2}d_{4}^{2}-176b_{0}b_{6}d_{2}^{2}d_{4}\\ -176Q\;v^{2}b_{0}^{2}b_{2}d_{2}d_{8}-264Q\;v^{2}b_{0}^{2}b_{2}d_{4}d_{6}-132Q\;v^{2}b_{0}^{2}b_{4}d_{2}d_{6}-88Q\;v^{2}b_{0}^{2}b_{4}d_{4}^{2}-88Q\;v^{2}b_{0}^{2}b_{6}d_{2}d_{4}\\ -22Q\;v^{2}b_{0}^{2}b_{8}d_{2}^{2}-66Q\;v^{2}b_{0}b_{2}^{2}d_{2}d_{6}-44Q\;v^{2}b_{0}b_{2}^{2}d_{4}^{2}-88Q\;v^{2}b_{0}b_{2}b_{4}d_{2}d_{4}-22Q\;v^{2}b_{0}b_{2}b_{6}d_{2}^{2}\\ +4Qb_{2}^{2}d_{4}^{2}+8Qb_{2}b_{4}d_{2}d_{4}+2Qb_{2}b_{6}d_{2}^{2}+Qb_{4}^{2}d_{2}^{2}-11b_{0}^{2}b_{2}b_{8}d_{2}-11b_{0}^{2}b_{4}b_{6}d_{2}+297b_{0}^{2}c_{2}c_{8}d_{2}\\ +22Qvb_{0}b_{8}d_{2}^{2}+66Qvb_{2}^{2}d_{2}d_{6}+44Qvb_{2}^{2}d_{4}^{2}+88Qvb_{2}b_{4}d_{2}d_{4}+22Qvb_{2}b_{6}d_{2}^{2}+11Qvb_{4}^{2}d_{2}^{2}\\ +160Qvb_{0}^{2}b_{2}d_{2}d_{8}+240Qvb_{0}^{2}b_{2}d_{4}d_{6}+120Qvb_{0}^{2}b_{4}d_{2}d_{6}+80Qvb_{0}^{2}b_{4}d_{4}^{2}+80Qvb_{0}^{2}b_{6}d_{2}d_{4}\\ +Qb_{0}b_{4}^{2}d_{2}^{2}-286vb_{0}^{2}b_{2}c_{8}d_{2}-374vb_{0}^{2}b_{4}c_{6}d_{2}-374vb_{0}^{2}b_{6}c_{4}d_{2}-286vb_{0}^{2}b_{8}c_{2}d_{2}-2Q^{3}d_{2}d_{8}\\ +176Qvb_{0}b_{2}d_{2}d_{8}+264Qvb_{0}b_{2}d_{4}d_{6}+132Qvb_{0}b_{4}d_{2}d_{6}+88Qvb_{0}b_{4}d_{4}^{2}+88Qvb_{0}b_{6}d_{2}d_{4}\\ +8Qb_{0}^{2}b_{6}d_{2}d_{4}+2Qb_{0}^{2}b_{8}d_{2}^{2}+6Qb_{0}b_{2}^{2}d_{2}d_{6}+4Qb_{0}b_{2}^{2}d_{4}^{2}+8Qb_{0}b_{2}b_{4}d_{2}d_{4}+2Qb_{0}b_{2}b_{6}d_{2}^{2}\\ +16Qb_{0}^{3}d_{4}d_{8}+9Qb_{0}^{3}d_{6}^{2}+16Qb_{0}^{2}b_{2}d_{2}d_{8}+24Qb_{0}^{2}b_{2}d_{4}d_{6}+12Qb_{0}^{2}b_{4}d_{2}d_{6}+8Qb_{0}^{2}b_{4}d_{4}^{2}\\ -22Q^{3}vd_{2}d_{8}-33Q^{3}vd_{4}d_{6}-2Q^{3}b_{0}d_{2}d_{8}-3Q^{3}b_{0}d_{4}d_{6}+176Qvb_{0}^{2}d_{4}d_{8}+99Qvb_{0}^{2}d_{6}^{2}\\ +24Qb_{0}b_{2}d_{4}d_{6}+12Qb_{0}b_{4}d_{2}d_{6}+8Qb_{0}b_{4}d_{4}^{2}+8Qb_{0}b_{6}d_{2}d_{4}+2Qb_{0}b_{8}d_{2}^{2}+6Qb_{2}^{2}d_{2}d_{6}\\ +20Qvb_{0}^{2}b_{8}d_{2}^{2}+60Qvb_{0}b_{2}^{2}d_{2}d_{6}+40Qvb_{0}b_{2}^{2}d_{4}^{2}+80Qvb_{0}b_{2}b_{4}d_{2}d_{4}+20Qvb_{0}b_{2}b_{6}d_{2}^{2}\\ +10Qvb_{0}b_{4}^{2}d_{2}^{2}+297v^{2}b_{0}^{2}b_{2}b_{8}d_{2}+385v^{2}b_{0}^{2}b_{4}b_{6}d_{2}-11v^{2}b_{0}^{2}c_{2}c_{8}d_{2}-11v^{2}b_{0}^{2}c_{4}c_{6}d_{2}\\ -3Q^{3}d_{4}d_{6}+44Q^{2}d_{2}^{2}d_{8}+66Q^{2}d_{2}d_{4}d_{6}+16Qb_{0}^{2}d_{4}d_{8}+9Qb_{0}^{2}d_{6}^{2}+16Qb_{0}b_{2}d_{2}d_{8}\\ -11Q\;v^{2}b_{0}b_{4}^{2}d_{2}^{2}-20Q^{3}vb_{0}d_{2}d_{8}-30Q^{3}vb_{0}d_{4}d_{6}+160Qvb_{0}^{3}d_{4}d_{8}+90Qvb_{0}^{3}d_{6}^{2}\\ -44b_{0}b_{8}d_{2}^{3}-132b_{2}^{2}d_{2}^{2}d_{6}-88b_{2}^{2}d_{2}d_{4}^{2}-176b_{2}b_{4}d_{2}^{2}d_{4}-44b_{2}b_{6}d_{2}^{3}-22b_{4}^{2}d_{2}^{3}) \end{array},$$

$$\begin{array}{l} c_{12}=\frac{1}{672b_{0}^{2}d_{2}\left(vb_{0}-b_{0}-1\right)}(130Q^{3}v^{2}b_{0}d_{2}d_{10}+208Q^{3}v^{2}b_{0}d_{4}d_{8}+117Q^{3}v^{2}b_{0}d_{6}^{2}-108Q^{3}vb_{0}d_{6}^{2}\\ -936Q\;v^{2}b_{0}^{2}b_{2}d_{6}^{2}-832Q\;v^{2}b_{0}^{2}b_{4}d_{2}d_{8}-1248Q\;v^{2}b_{0}^{2}b_{4}d_{4}d_{6}-624Q\;v^{2}b_{0}^{2}b_{6}d_{2}d_{6}-416Q\;v^{2}b_{0}^{2}b_{6}d_{4}^{2}\\ +1040Qvb_{0}^{2}d_{4}d_{10}+1248Qvb_{0}^{2}d_{6}d_{8}+1040Qvb_{0}b_{2}d_{2}d_{10}+1664Qvb_{0}b_{2}d_{4}d_{8}+936Qvb_{0}b_{2}d_{6}^{2}\\ +384Qvb_{0}b_{2}b_{6}d_{2}d_{4}+96Qvb_{0}b_{2}b_{8}d_{2}^{2}+192Qvb_{0}b_{4}^{2}d_{2}d_{4}+96Qvb_{0}b_{4}b_{6}d_{2}^{2}+1716v^{2}b_{0}^{2}b_{2}b_{10}d_{2}\\ +1152Qvb_{0}^{3}d_{6}d_{8}+960Qvb_{0}^{2}b_{2}d_{2}d_{10}+1536Qvb_{0}^{2}b_{2}d_{4}d_{8}+864Qvb_{0}^{2}b_{2}d_{6}^{2}+768Qvb_{0}^{2}b_{4}d_{2}d_{8}\\ -208Q\;v^{2}b_{0}b_{4}^{2}d_{2}d_{4}-104Q\;v^{2}b_{0}b_{4}b_{6}d_{2}^{2}-120Q^{3}vb_{0}d_{2}d_{10}-192Q^{3}vb_{0}d_{4}d_{8}+960Qvb_{0}^{3}d_{4}d_{10}\\ -832b_{0}b_{6}d_{2}d_{4}^{2}-832b_{0}b_{8}d_{2}^{2}d_{4}-208b_{0}b_{10}d_{2}^{3}-832b_{2}^{2}d_{2}^{2}d_{8}-1248b_{2}^{2}d_{2}d_{4}d_{6}-1248b_{2}b_{4}d_{2}^{2}d_{6}\\ +96Qb_{0}^{2}b_{4}d_{4}d_{6}+48Qb_{0}^{2}b_{6}d_{2}d_{6}+32Qb_{0}^{2}b_{6}d_{4}^{2}+32Qb_{0}^{2}b_{8}d_{2}d_{4}+8Qb_{0}^{2}b_{10}d_{2}^{2}+32Qb_{0}b_{2}^{2}d_{2}d_{8}\\ +832Qvb_{0}b_{4}d_{2}d_{8}+1248Qvb_{0}b_{4}d_{4}d_{6}+624Qvb_{0}b_{6}d_{2}d_{6}+416Qvb_{0}b_{6}d_{4}^{2}+416Qvb_{0}b_{8}d_{2}d_{4}\\ -52b_{0}^{2}b_{4}b_{8}d_{2}-26b_{0}^{2}b_{6}^{2}d_{2}+1716b_{0}^{2}c_{2}c_{10}d_{2}+2340b_{0}^{2}c_{4}c_{8}d_{2}+1274b_{0}^{2}c_{6}^{2}d_{2}-2080b_{0}b_{2}d_{2}^{2}d_{10}\\ +1152Qvb_{0}^{2}b_{4}d_{4}d_{6}+576Qvb_{0}^{2}b_{6}d_{2}d_{6}+384Qvb_{0}^{2}b_{6}d_{4}^{2}+384Qvb_{0}^{2}b_{8}d_{2}d_{4}+96Qvb_{0}^{2}b_{10}d_{2}^{2}\\ -3328b_{0}b_{2}d_{2}d_{4}d_{8}-1872b_{0}b_{2}d_{2}d_{6}^{2}-1664b_{0}b_{4}d_{2}^{2}d_{8}-2496b_{0}b_{4}d_{2}d_{4}d_{6}-1248b_{0}b_{6}d_{2}^{2}d_{6}\\ +32Qb_{0}b_{6}d_{4}^{2}+32Qb_{0}b_{8}d_{2}d_{4}+8Qb_{0}b_{10}d_{2}^{2}+32Qb_{2}^{2}d_{2}d_{8}+48Qb_{2}^{2}d_{4}d_{6}+48Qb_{2}b_{4}d_{2}d_{6}\\ -2288vb_{0}^{2}b_{8}c_{4}d_{2}-1664vb_{0}^{2}b_{10}c_{2}d_{2}-10Q^{3}d_{2}d_{10}-16Q^{3}d_{4}d_{8}-9Q^{3}d_{6}^{2}+260Q^{2}d_{2}^{2}d_{10}\\ -130Q^{3}vd_{2}d_{10}-208Q^{3}vd_{4}d_{8}-117Q^{3}vd_{6}^{2}-10Q^{3}b_{0}d_{2}d_{10}-16Q^{3}b_{0}d_{4}d_{8}-9Q^{3}b_{0}d_{6}^{2}\\ +104Qvb_{0}b_{10}d_{2}^{2}+416Qvb_{2}^{2}d_{2}d_{8}+624Qvb_{2}^{2}d_{4}d_{6}+624Qvb_{2}b_{4}d_{2}d_{6}+416Qvb_{2}b_{4}d_{4}^{2}\\ +32Qb_{2}b_{4}d_{4}^{2}+32Qb_{2}b_{6}d_{2}d_{4}+8Qb_{2}b_{8}d_{2}^{2}+16Qb_{4}^{2}d_{2}d_{4}+8Qb_{4}b_{6}d_{2}^{2}-52b_{0}^{2}b_{2}b_{10}d_{2}\\ +416Qvb_{2}b_{6}d_{2}d_{4}+104Qvb_{2}b_{8}d_{2}^{2}+208Qvb_{4}^{2}d_{2}d_{4}+104Qvb_{4}b_{6}d_{2}^{2}+80Qb_{0}^{3}d_{4}d_{10}\\ +48Qb_{0}b_{2}^{2}d_{4}d_{6}+48Qb_{0}b_{2}b_{4}d_{2}d_{6}+32Qb_{0}b_{2}b_{4}d_{4}^{2}+32Qb_{0}b_{2}b_{6}d_{2}d_{4}+8Qb_{0}b_{2}b_{8}d_{2}^{2}\\ +2340v^{2}b_{0}^{2}b_{4}b_{8}d_{2}+1274v^{2}b_{0}^{2}b_{6}^{2}d_{2}-52v^{2}b_{0}^{2}c_{2}c_{10}d_{2}-52v^{2}b_{0}^{2}c_{4}c_{8}d_{2}-26v^{2}b_{0}^{2}c_{6}^{2}d_{2}\\ -624Q\;v^{2}b_{0}b_{2}b_{4}d_{2}d_{6}-416Q\;v^{2}b_{0}b_{2}b_{4}d_{4}^{2}-416Q\;v^{2}b_{0}b_{2}b_{6}d_{2}d_{4}-104Q\;v^{2}b_{0}b_{2}b_{8}d_{2}^{2}\\ -1040Q\;v^{2}b_{0}^{3}d_{4}d_{10}-1248Q\;v^{2}b_{0}^{3}d_{6}d_{8}-1040Q\;v^{2}b_{0}^{2}b_{2}d_{2}d_{10}-1664Q\;v^{2}b_{0}^{2}b_{2}d_{4}d_{8}\\ +16Qb_{0}b_{4}^{2}d_{2}d_{4}8Qb_{0}b_{4}b_{6}d_{2}^{2}-1664vb_{0}^{2}b_{2}c_{10}d_{2}-2288vb_{0}^{2}b_{4}c_{8}d_{2}-2496vb_{0}^{2}b_{6}c_{6}d_{2}\\ +128Qb_{0}b_{2}d_{4}d_{8}+72Qb_{0}b_{2}d_{6}^{2}+64Qb_{0}b_{4}d_{2}d_{8}+96Qb_{0}b_{4}d_{4}d_{6}+48Qb_{0}b_{6}d_{2}d_{6}\\ +96Qb_{0}^{3}d_{6}d_{8}+80Qb_{0}^{2}b_{2}d_{2}d_{10}+128Qb_{0}^{2}b_{2}d_{4}d_{8}+72Qb_{0}^{2}b_{2}d_{6}^{2}+64Qb_{0}^{2}b_{4}d_{2}d_{8}\\ -416Q\;v^{2}b_{0}^{2}b_{8}d_{2}d_{4}-104Q\;v^{2}b_{0}^{2}b_{10}d_{2}^{2}-416Q\;v^{2}b_{0}b_{2}^{2}d_{2}d_{8}-624Q\;v^{2}b_{0}b_{2}^{2}d_{4}d_{6}\\ +384Qvb_{0}b_{2}^{2}d_{2}d_{8}+576Qvb_{0}b_{2}^{2}d_{4}d_{6}+576Qvb_{0}b_{2}b_{4}d_{2}d_{6}+384Qvb_{0}b_{2}b_{4}d_{4}^{2}\\ +416Q^{2}d_{2}d_{4}d_{8}+234Q^{2}d_{2}d_{6}^{2}+80Qb_{0}^{2}d_{4}d_{10}+96Qb_{0}^{2}d_{6}d_{8}+80Qb_{0}b_{2}d_{2}d_{10}\\ -832b_{2}b_{4}d_{2}d_{4}^{2}-832b_{2}b_{6}d_{2}^{2}d_{4}-208b_{2}b_{8}d_{2}^{3}-416b_{4}^{2}d_{2}^{2}d_{4}-208b_{4}b_{6}d_{2}^{3}) \end{array},$$

$$d_{2}=-\frac{Q}{4b_{0}},$$

$$d_{4}=\frac{d_{2}\left(Q^{2}-8b_{0}b_{2}\right)}{16b_{0}^{2}},$$

$$d_{6}=\frac{Q^{2}d_{2}d_{4}-4b_{0}^{2}d_{4}^{2}-8b_{0}b_{2}d_{2}d_{4}-2b_{0}b_{4}d_{2}^{2}-b_{2}^{2}d_{2}^{2}}{6b_{0}^{2}d_{2}},$$

$$\begin{array}{l} d_{8}=\frac{1}{16b_{0}^{2}d_{2}}(3Q^{2}d_{2}d_{6}+2Q^{2}d_{4}^{2}-24b_{0}^{2}d_{4}d_{6}-24b_{0}b_{2}d_{2}d_{6}-16b_{0}b_{2}d_{4}^{2}-16b_{0}b_{4}d_{2}d_{4}\\ -4b_{0}b_{6}d_{2}^{2}-8b_{2}^{2}d_{2}d_{4}-4b_{2}b_{4}d_{2}^{2}) \end{array},$$

$$\begin{array}{l} d_{10}=\frac{1}{10b_{0}^{2}d_{2}}(2Q^{2}d_{2}d_{8}+3Q^{2}d_{4}d_{6}-16b_{0}^{2}d_{4}d_{8}-9b_{0}^{2}d_{6}^{2}-16b_{0}b_{2}d_{2}d_{8}-24b_{0}b_{2}d_{4}d_{6}\\ -12b_{0}b_{4}d_{2}d_{6}-8b_{0}b_{4}d_{4}^{2}-8b_{0}b_{6}d_{2}d_{4}-2b_{0}b_{8}d_{2}^{2}-6b_{2}^{2}d_{2}d_{6}-4b_{2}^{2}d_{4}^{2}-8b_{2}b_{4}d_{2}d_{4}\\ -2b_{2}b_{6}d_{2}^{2}-b_{4}^{2}d_{2}^{2}) \end{array},$$

$$\begin{array}{l} d_{12}=\frac{1}{48b_{0}^{2}d_{2}}(10Q^{2}d_{2}d_{10}+16Q^{2}d_{4}d_{8}+9Q^{2}d_{6}^{2}-80b_{0}^{2}d_{4}d_{10}-96b_{0}^{2}d_{6}d_{8}-80b_{0}b_{2}d_{2}d_{10}\\ -128b_{0}b_{2}d_{4}d_{8}-72b_{0}b_{2}d_{6}^{2}-64b_{0}b_{4}d_{2}d_{8}-96b_{0}b_{4}d_{4}d_{6}-48b_{0}b_{6}d_{2}d_{6}-32b_{0}b_{6}d_{4}^{2}\\ -32b_{0}b_{8}d_{2}d_{4}-8b_{0}b_{10}d_{2}^{2}-32b_{2}^{2}d_{2}d_{8}-48b_{2}^{2}d_{4}d_{6}-48b_{2}b_{4}d_{2}d_{6}-32b_{2}b_{4}d_{4}^{2}\\ -32b_{2}b_{6}d_{2}d_{4}-8b_{2}b_{8}d_{2}^{2}-16b_{4}^{2}d_{2}d_{4}-8b_{4}b_{6}d_{2}^{2}) \end{array}.$$
